# Straightforward preparation of highly loaded MWCNT–polyamine hybrids and their application in catalysis†

**DOI:** 10.1039/d0na00291g

**Published:** 2020-07-14

**Authors:** Vincenzo Campisciano, René Burger, Carla Calabrese, Leonarda Francesca Liotta, Paolo Lo Meo, Michelangelo Gruttadauria, Francesco Giacalone

**Affiliations:** Department of Biological, Chemical and Pharmaceutical Sciences and Technologies, University of Palermo Viale delle Scienze, Ed. 17 90128 Palermo Italy michelangelo.gruttadauria@unipa.it francesco.giacalone@unipa.it; Department of Natural Sciences, Bonn-Rhein-Sieg University of Applied Sciences von-Liebig-Strasse 20 D-53359 Rheinbach Germany; Istituto per lo Studio dei Materiali Nanostrutturati ISMN-CNR Via Ugo La Malfa, 153 90146 Palermo Italy

## Abstract

Multiwalled carbon nanotubes (MWCNTs) were easily and efficiently functionalised with highly cross-linked polyamines. The radical polymerisation of two bis-vinylimidazolium salts in the presence of pristine MWCNTs and azobisisobutyronitrile (AIBN) as a radical initiator led to the formation of materials with a high functionalisation degree. The subsequent treatment with sodium borohydride gave rise to the reduction of imidazolium moieties with the concomitant formation of secondary and tertiary amino groups. The obtained materials were characterised by thermogravimetric analysis (TGA), elemental analysis, solid state ^13^C-NMR, Fourier-transform infrared spectroscopy (FT-IR), transmission electron microscopy (TEM), potentiometric titration, and temperature programmed desorption of carbon dioxide (CO_2_-TPD). One of the prepared materials was tested as a heterogeneous base catalyst in C–C bond forming reactions such as the Knoevenagel condensation and Henry reaction. Furthermore, two examples concerning a sequential one-pot approach involving two consecutive reactions, namely Knoevenagel and Michael reactions, were reported.

## Abstract 2

Two MWCNT–polyamine hybrids were prepared, fully characterised, and used as catalysts for different C–C bond forming reactions.

## Graphical abstract

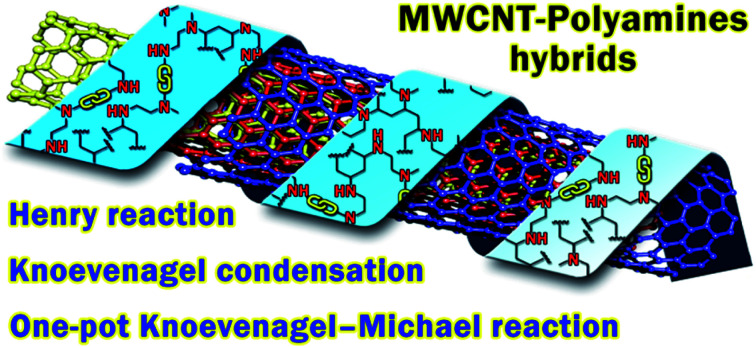

## Introduction

The outstanding electrical and mechanical features of carbon nanotubes (CNTs) are well known and explain why CNT-based materials have aroused significant interest for use in plenty of applications such as energy storage,^1–3^ electronics,^4–6^ catalysis,^7–9^ structural reinforcement,^3,10,11^ and nanomedicine,^12–15^ among others. However, pristine CNTs suffer from some inherent drawbacks mainly related to limited dispersibility that reduce their processability. Therefore, it is not surprising that many research efforts have been made aimed at efficiently functionalising CNTs both to improve their handling and to impart other specific features. It is possible to find a large number of examples concerning the noncovalent and covalent functionalisation of CNTs.^16–18^ Among these routes, the one directed towards the covalent modification of CNTs can ensure both a good extent of functionalisation and an improved dispersibility in different media. Therefore, focusing on the covalent modification approaches, two methods were developed, namely the oxidation of CNTs followed by their amidation or esterification and the direct addition reactions at the CNTs’ sidewalls.^16,19–21^

The first functionalisation method of CNTs consists of their initial oxidation. Depending on the strength of the oxidative/acid treatment of pristine CNTs, nanotube fragmentation along with their decoration with various oxygenated functional groups (carboxyl, carbonyl, hydroxyl, *etc.*) can be obtained. However, shortening of the pristine CNTs can be minimized by adopting milder conditions, such as the use of refluxing nitric acid, preserving their electronic and mechanical properties. The second step involves the post-functionalisation of the primary oxygenated functional groups added onto the surface of CNTs by means of standard amidation and esterification reactions.

On the other hand, the direct functionalisation of CNTs allows us to exploit the π-conjugated carbon framework of nanotubes avoiding any pre-treatment of the pristine CNTs. In the first case, the post-functionalisation with amide and ester groups depends only on the amount of the primary oxygenated functional groups inserted onto the CNTs. Conversely, the direct functionalisation methods require high reactivity of the species involved. This means that the functionalisation could be initiated mainly in the highly reactive areas of CNTs (the most curved regions or those close to defects formed during their synthesis) from where it can continue further.

It is well known that any functionalisation method of CNTs will become more and more interesting and convenient if it refers more to some fundamental principles. As a matter of fact, an ideal and highly appealing functionalisation process should be simple, efficient, less time-consuming and easily scalable. Only in this way, the demand for great amounts of highly functionalised CNTs, which could be crucial for some specific applications, can be met. In this scenario, due to the high radical accepting capacity of CNTs which makes them good radical scavengers^22–26^ and anti-oxidants,^27–29^ a very useful technique able to give rise to high functionalisation degrees could be the free-radical modification of CNTs. This modification approach results in less damage of pristine nanotubes’ sidewalls and in CNT debundling, as a result of their functionalisation and better dispersion/solubility and processability. Moreover, free-radical modification allows for the direct functionalisation of pristine CNTs avoiding any pre-treatment, as evidenced by several examples in which free-radical modification of the CNT surface led to the full coverage of the carbonaceous skeleton, in most cases.^30–42^

Among the different covalent modification approaches, those aimed at introducing amino groups on the surface of CNTs have raised growing interest. CNTs have been modified by means of grafting of amino group based polymers or small organic molecules, as shown by the large number of examples concerning the covalent modification of CNTs with hyperbranched^43–45^ or cross-linked polymers,^46–48^ polyamidoamine (PAMAM) dendrimers,^49–53^ and polyamines,^54–56^ among others.^30,31,57–59^ The application fields of amino-modified CNTs are numerous, as a matter of fact, some examples concerning the possible use of amino-functionalised CNTs involve, and are not limited to, their application as reinforcing agents in epoxy composites,^60–62^ solid base catalysts for organic transformations,^9,63–65^ CO_2_ adsorbents,^66–71^ supports for metal nanoparticle immobilization,^53,72–75^ metal scavengers,^47,48,56,58^ and gene or drug delivery systems.^50,52,59,76–81^ Ensuring a suitable amino group content of CNTs certainly represents a key point regardless of the purpose of use of the prepared material. Therefore, the development of an easily feasible synthetic method to obtain highly amino-functionalised CNT materials represents an important goal.

We have recently reported a simple functionalisation method of multi-walled carbon nanotubes (MWCNTs) in which the nanotubes acted as a sort of templating agent for the polymerisation of a highly cross-linked imidazolium network, resulting in a cylindrical coating around the nanotube skeleton.^82^ This straightforward approach, which consisted of one-step radical polymerisation of a bis-vinylimidazolium salt in the presence of nanotubes, allowed obtaining the direct and efficient functionalisation of pristine MWCNTs with a large amount of polyimidazolium salt, since the final content of polymerised network onto nanotubes was higher than 90 wt%.

In another study we showed that it was possible to easily obtain cross-linked polyamines, containing secondary and tertiary amines, by means of the reduction of the corresponding cross-linked imidazolium-based material with sodium borohydride.^83^ Given the extremely high versatility of amino-functionalised carbon-based materials, and in the context of the current interest aroused by the production of heterogeneous base catalysts, which can find application in the catalysis of some relevant C–C bond forming reactions such as Knoevenagel condensation and Henry reaction,^84–86^ herein we propose a new method for obtaining highly loaded cross-linked polyamine–MWCNT materials through the reduction of the corresponding easily accessible cross-linked polyimidazolium salt–MWCNT hybrids. More in detail, one of the prepared materials was not only tested as a heterogeneous base catalyst in the above-mentioned Knoevenagel and Henry reactions, but its use in two examples concerning a sequential one-pot approach involving two consecutive reactions, namely Knoevenagel and Michael reactions, is also reported.

## Experimental

### Materials and methods

Chemicals and solvents were purchased from commercial suppliers and used as received without further purification. Thermogravimetric analysis (TGA) was performed under nitrogen flow from 100 to 1000 °C with a heating rate of 10 °C min^−1^ with a Mettler Toledo TGA/DSC STAR System. The temperature was increased starting from rt up to 100 °C, and then this temperature was maintained for 30 minutes to remove adsorbed water before reaching 1000 °C. The CHN combustion elemental analysis was performed on a Perkin Elmer 2400 Series II analyser. ^13^C CP-MAS NMR spectra were acquired on a Bruker Advance II 400 spectrometer operating at 100.63 MHz for ^13^C nuclides and 400.15 MHz for ^1^H nuclides equipped with a 4 mm (H–X) double channel CP-MAS probe. The samples were placed in a 4 mm zirconia rotor closed with Kel-F caps. The spectra were measured at a MAS speed of 8 kHz, with 2 ms contact time, 3 s delay time and an excitation pulse of 4.7 μs on the ^1^H nucleus, and 1024 scans were performed. The Hartmann–Hahn-condition was optimized using an adamantane standard sample, which was also used as an external chemical shift reference. FT-IR spectra (KBr disk) were recorded on an Agilent Technologies Cary 630 FT-IR spectrometer. Transmission electron microscopy (TEM) images were recorded on a Philips TECNAI 10 microscope at 80 kV. The potentiometric reverse acid–base titration was performed by the addition of known volumes (2.5–40 μL steps) of a standard 1 M NaOH solution (1 mL) to an aqueous suspension (14 mL of distilled water and 1 mL of methanol) of the reduced material (*ca.* 25 mg) treated with an excess of 0.1 M HCl (5 mL; *n*_HCl_ = 0.5 mmol). The obtained suspension was stirred overnight at room temperature and bubbled with argon for 20 minutes prior to the start of the titration. The aqueous NaOH solution was added using a Chemetron microliter syringe while measuring the pH value with a Crison micro pH 2001 system. Temperature-programmed desorption (CO_2_-TPD) was carried out using a Micromeritics Autochem 2950HP apparatus equipped with a thermal conductivity detector (TCD) and an IR analyser (ABB Uras 14). The sample (200 mg) was put in a quartz U shaped reactor with an inner diameter of 12 mm, electrically heated in a furnace. The adsorption of CO_2_ was performed at 30 °C under pure CO_2_ (50 mL min^−1^) for 1 h, and then He (50 mL min^−1^) was used to remove the physically adsorbed CO_2_ by purging for 1 h at room temperature. Finally, the temperature-programmed desorption procedure was carried out under He flow (50 mL min^−1^) from 30 °C to 150 °C (heating rate of 4 °C min^−1^), holding at 150 °C for 30 min. ^1^H NMR spectra of the products of all the catalytic tests were recorded on a Bruker 300 MHz spectrometer.

### Synthesis of bis-vinylimidazolium salts 1a and 1b

Bis-vinylimidazolium salts 1a and 1b were synthesized following a reported procedure with minor changes.^87^

#### Synthesis of compound 1a

A solution of 1,4-dibromobutane (0.75 mL, 6.22 mmol) and 1-vinylimidazole (1.20 mL, 12.99 mmol) in chloroform (5.5 mL) was stirred at 50 °C for 20 h. A white precipitate was obtained and chloroform was removed by simple decantation. Afterwards, methanol was added and gently warmed to solubilize the solid obtaining a yellowish viscous oil, which after the addition of diethyl ether and sonication gave rise to a white precipitate. Diethyl ether was then removed and the whole procedure of solubilization in methanol and precipitation with diethyl ether was repeated twice. The obtained white solid was dried under vacuum at 40 °C. Bis-vinylimidazolium salt 1a was obtained as a white solid (2.332 g; 93%).

#### Synthesis of compound 1b

A solution of 1,4-dibromo-*p*-xylene (1.5 g, 5.57 mmol) and 1-vinylimidazole (1.10 mL, 11.91 mmol) in toluene (5.5 mL) was stirred at 90 °C for 20 h. For the purification of 1b the same procedure reported for 1a was followed. Compound 1b was obtained as a white solid (2.263 g; 90%).

### Preparation of materials Imi-But-MWCNT and Imi-Xyl-MWCNT

In a two-neck round bottom flask, 1.67 mmol of bis-vinylimidazolium salt (674 mg for 1a; 754 mg for 1b), 40 mg of MWCNTs and 15 mL of absolute ethanol were mixed. The suspension was sonicated for 25 min to achieve a fine dispersion of the nanotubes. Under the protection of an argon atmosphere, AIBN (29 mg, 0.17 mmol) was added. After bubbling the reaction mixture with argon for 15 min, it was refluxed at 80 °C under stirring for 24 h. The reaction mixture was allowed to reach room temperature and 20 mL of methanol were added to the flask. The dark suspension was transferred into a centrifuge tube and sonicated for several minutes before centrifugation at 5000 rpm for 5 min. The clear supernatant was removed, and the dark residue was washed twice with methanol and once with diethyl ether by sonication and centrifugation. The product was dried under vacuum overnight at 40 °C in a glass oven. Imi-But-MWCNT was obtained as a dark grey solid (549 mg), CHN elemental analysis (%): C, 43.77; H, 5.13; N, 10.97. Imi-Xyl-MWCNT was obtained as a dark grey solid (713 mg), CHN elemental analysis (%): C, 47.21; H, 4.71; N, 10.20.

### Preparation of materials NH-But-MWCNT and NH-Xyl-MWCNT

In a round bottom flask, 500 mg of Imi-But-MWCNT or 560 mg of Imi-Xyl-MWCNT and 18 mL of absolute ethanol were added. The mixture was sonicated for 20 min before adding 990 mg and 1025 mg of NaBH_4_ for Imi-But-MWCNT and Imi-Xyl-MWCNT, respectively. The reaction mixture was refluxed at 80 °C under an argon atmosphere for 22 h. The reaction mixture was allowed to reach room temperature and 20 mL of methanol were added. The dark suspension was transferred into a centrifuge tube, sonicated for several minutes before centrifugation at 5000 rpm for 10 min and the supernatant was removed. Two different procedures were adopted for the following washings of the two materials. In the case of NH-But-MWCNT, it was suspended and sonicated in water before vacuum filtration using a Durapore® PVDF membrane filter (VVLP, hydrophilic PVDF, 0.1 μm pore size). The residue was thoroughly washed with water and dried overnight under vacuum at 40 °C in a glass oven. Material NH-But-MWCNT was obtained as a black powder (299 mg); CHN elemental analysis (%): C, 62.18; H, 8.69; N, 15.52. For NH-Xyl-MWCNT the further washings were carried out by sonication and subsequent centrifugation twice with water, once with methanol and once with diethyl ether before drying the residue overnight under vacuum at 40 °C in a glass oven. Material NH-Xyl-MWCNT was obtained as a black powder (358 mg); CHN elemental analysis (%): C, 67.51; H, 7.65; N, 14.47.

### General procedure for the Knoevenagel condensation

In a glass vial with a screw cap, 1 mmol of aldehyde, 1 mmol of ethyl cyanoacetate, 1.58 mg of NH-But-MWCNT, and 150 μL of ethanol were added. The mixture was stirred at 30 °C for the specified time before dilution by addition of DCM and filtration for catalyst removal. The filtrate was evaporated under vacuum at 40 °C and the residue was weighed and analysed by ^1^H NMR spectroscopy.

### General recycling procedure for the Knoevenagel condensation

In a glass vial with a screw cap, 2 mmol of aldehyde, 2 mmol of ethyl cyanoacetate, 3.16 mg of NH-But-MWCNT (2 mol%), and 300 μL of ethanol were added and stirred at 30 °C for the specified time. The reaction mixture was diluted by the addition of CHCl_3_/Et_2_O 4 : 1 (v/v), sonicated for several minutes, and centrifuged at 5000 rpm for 5 min. The supernatant was recovered and the washing procedure was repeated twice with CHCl_3_/Et_2_O 4 : 1 (v/v) and finally with Et_2_O. The recovered catalyst was dried at 50 °C and then reused. The collected supernatants were evaporated under vacuum at 40 °C and the residue was weighed and analysed by ^1^H NMR spectroscopy. The catalytic tests at 1 mol% loading were carried out on a scale of 1.0 mmol. For the reactivation of the catalyst, reused NH-But-MWCNT was treated with 200 μL of formic acid and the obtained suspension was stirred at room temperature for 2 h. The mixture was diluted with Et_2_O, sonicated for several minutes, and centrifuged at 5000 rpm for 5 min. The supernatant was removed and the whole washing procedure was repeated once with Et_2_O. Then a solution of 50 vol% NEt_3_ in THF was added and the resulting suspension was stirred at room temperature for 1 h, centrifuged at 5000 rpm for 5 min, and the supernatant was removed. The washing procedure described above was repeated twice with THF and once with Et_2_O. The catalyst was dried at 50 °C and then reused.

### General procedure for the sequential one-pot Knoevenagel–Michael reaction

In a glass vial with a screw cap, 2.5 mmol of aldehyde, 2.5 mmol of ethyl cyanoacetate, 3.94 mg of NH-But-MWCNT (2 mol%), and 375 μL of ethanol were added. The mixture was stirred at room temperature for the specified time. Then, 25 mmol of nitromethane were added and the reaction mixture was stirred at room temperature for the specified time. The mixture was diluted by the addition of DCM and filtered for catalyst removal. The filtrate was evaporated under vacuum at 40 °C and the residue was weighed and analysed by ^1^H NMR spectroscopy.

### General procedure for the Henry reaction

In a glass vial with a screw cap, 0.254 mmol of aldehyde, 2.54 mmol of nitromethane, 1 mg of NH-But-CNT (5 mol%), and 345 μL of ethanol were added. The mixture was stirred at room temperature for 24 h before dilution by the addition of DCM and filtration for catalyst removal. The filtrate was evaporated under vacuum at 40 °C and the residue was weighed and analysed by ^1^H NMR spectroscopy.

## Results and discussion

**Scheme 1:**
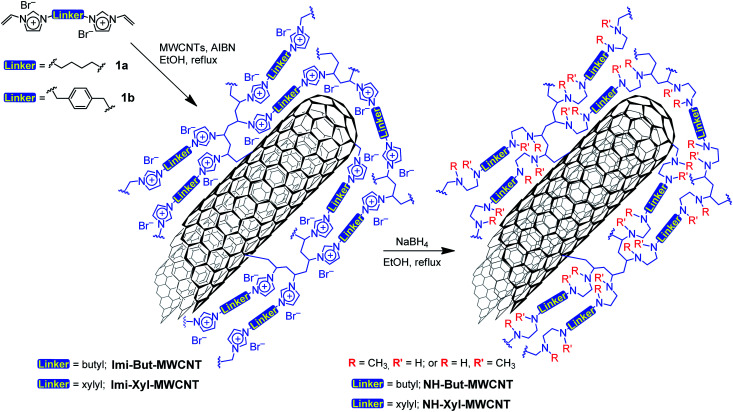
Preparation of materials NH-But-MWCNT and NH-Xyl-MWCNT.

Two bis-vinylimidazolium salts (1a,b) were used for the direct functionalisation of pristine MWCNTs by means of free-radical polymerisation initiated by azobisisobutyronitrile (AIBN), as reported in Scheme 1. The as-prepared materials Imi-But-MWCNT and Imi-Xyl-MWCNT were reduced with sodium borohydride in refluxing ethanol leading to the opening of the imidazolium rings and the subsequent formation of secondary and tertiary amino groups. Following this synthetic strategy, it was possible to obtain the corresponding highly cross-linked polyamine materials NH-But-MWCNT and NH-Xyl-MWCNT (Scheme 1).

**Fig. 1:**
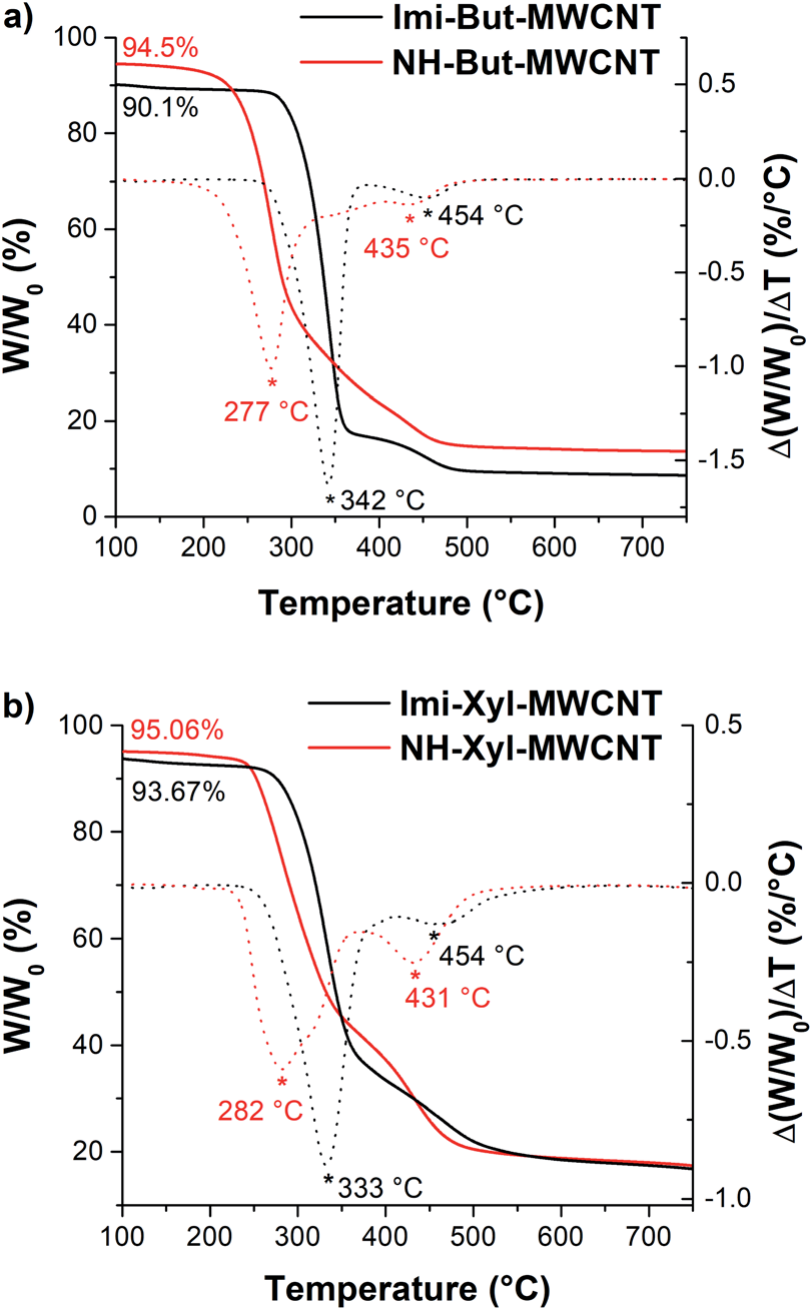
TGA (solid lines) and DTG (dotted lines) of (a) Imi-But-MWCNT and NH-But-MWCNT and (b) Imi-Xyl-MWCNT and NH-Xyl-MWCNT. All the samples were thermostated at 100 °C for 30 minutes (see the Experimental section). The weights at 100 °C were not normalized to 100% to show the different moisture contents of the materials.

Materials NH-But-MWCNT and NH-Xyl-MWCNT were subjected to thermogravimetric analysis (TGA) to study both their thermal stability and the functionalisation degree of MWCNTs (Fig. 1). TGA was performed under a nitrogen atmosphere and no decomposition of MWCNTs up to 750 °C was observed; therefore, all the functionalisation degrees were calculated at 700 °C, at which temperature all the organic coatings around the nanotubes are expected to be totally decomposed. By comparing the thermogravimetric and derivative thermogravimetric (DTG) curves of Imi-But-MWCNT and NH-But-MWCNT (Fig. 1a), three remarks could be made. (i) Imi-But-MWCNT showed a higher thermal stability than the corresponding reduced material NH-But-MWCNT, since the degradation process of the two materials started at about 275 °C and 200 °C, respectively. The higher degradation temperature of Imi-But-MWCNT could be explained by the higher stability of the imidazolium moieties with respect to the secondary and tertiary amines present in material NH-But-MWCNT. (ii) The DTG curves (dotted lines in Fig. 1a) clearly showed the different degradation profiles of materials Imi-But-MWCNT and NH-But-MWCNT. It was possible to identify two degradation peaks for each material centred at 342 °C and 454 °C in the case of Imi-But-MWCNT, and at 277 °C and 435 °C for NH-But-MWCNT. (iii) The larger residual weight of NH-But-MWCNT (amino group content: 12.69 mmol g^−1^) at 700 °C was ascribed to the weight loss of the polymeric coating (*ca.* 37 wt% of the initial weight of 1a polymerised onto MWCNTs) due to the loss of two bromide anions and addition of ten hydrogen atoms per monomer unit after the reduction process of Imi-But-MWCNT (nitrogen content: 8.04 mmol g^−1^) with sodium borohydride.

In the case of materials Imi-Xyl-MWCNT and NH-Xyl-MWCNT, the analysis of thermogravimetric curves (Fig. 1b) confirmed the first two statements made for Imi-But-MWCNT and NH-But-MWCNT. As a matter of fact, TGA revealed that Imi-Xyl-MWCNT was stable up to 250 °C, unlike NH-But-MWCNT, which started to degrade at 225 °C. Moreover, as seen above, DTG curves (dotted lines in Fig. 1b) showed different profiles with two peaks centred at 333 °C and 454 °C for Imi-Xyl-MWCNT, and at 282 °C and 431 °C in the case of NH-Xyl-MWCNT. Despite the good outcome of the reduction reaction of Imi-Xyl-MWCNT, confirmed by solid state NMR and FT-IR analysis (see below), residual weights of Imi-Xyl-MWCNT and NH-Xyl-MWCNT at 700 °C were similar. Once again, a weight loss of the polymeric coating (*ca.* 33 wt% of the initial weight of 1b polymerised onto MWCNTs) after the reduction of Imi-Xyl-MWCNT with sodium borohydride could be expected. The elemental analysis data of materials Imi-Xyl-MWCNT and NH-Xyl-MWCNT (see the Experimental section) could provide a possible explanation for the absence of weight decrease (higher residual weight of NH-Xyl-MWCNT). As a matter of fact, elemental analysis data were in good agreement with the TGA results of NH-Xyl-MWCNT indicating an amino group content of 10.20 mmol g^−1^. In contrast, the TGA data seemed to underestimate the real functionalisation degree of Imi-Xyl-MWCNT determined by elemental analysis (nitrogen content: 7.28 mmol g^−1^). The reason for such a discrepancy in the case of Imi-Xyl-MWCNT could probably be the incomplete decomposition of the organic coating at 700 °C, whereas, after the reduction process, the organic coating of NH-Xyl-MWCNT was totally decomposed at the same temperature.

**Fig. 2:**
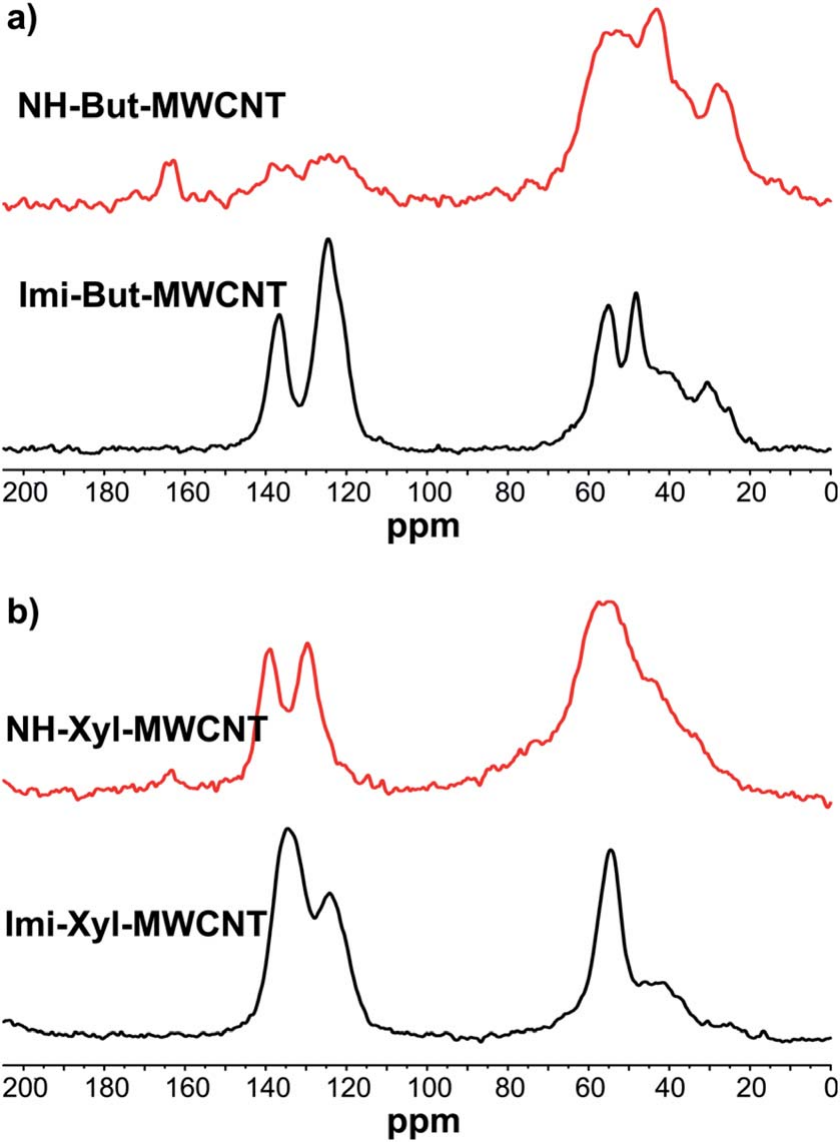
^13^C CP-MAS NMR of (a) Imi-But-MWCNT and NH-But-MWCNT and (b) Imi-Xyl-MWCNT and NH-Xyl-MWCNT.

The ^13^C cross-polarization magic angle spinning (^13^C CP-MAS) NMR spectra of all materials before (Imi-But-MWCNT and Imi-Xyl-MWCNT) and after (NH-But-MWCNT and NH-Xyl-MWCNT) the reduction with sodium borohydride were recorded (Fig. 2). In the solid-state NMR spectrum of Imi-But-MWCNT (Fig. 2a), two regions can be identified. In the first part of the spectrum (110–150 ppm region), the resonance of carbons belonging to the imidazolium units can be observed, whereas the carbons of the butyl chain fall in the second region around 50 ppm. The reduction process caused the almost complete disappearance of the signals attributed to the imidazolium moieties (Fig. 2a). In the case of the solid-state NMR spectra of Imi-Xyl-MWCNT and NH-Xyl-MWCNT (Fig. 2b), the same statements made above for materials with the butyl linker can be made. In this case, the signals in the 110–150 ppm region of NH-Xyl-MWCNT can be ascribed to the presence of the xylyl linker. However, it was possible to observe a marked intensity decrease of these signals, against a raised signal intensity in the region around 50 ppm due to the increase of the aliphatic contribution after the reduction and ring opening of the imidazolium moieties (Fig. 2b).

**Fig. 3:**
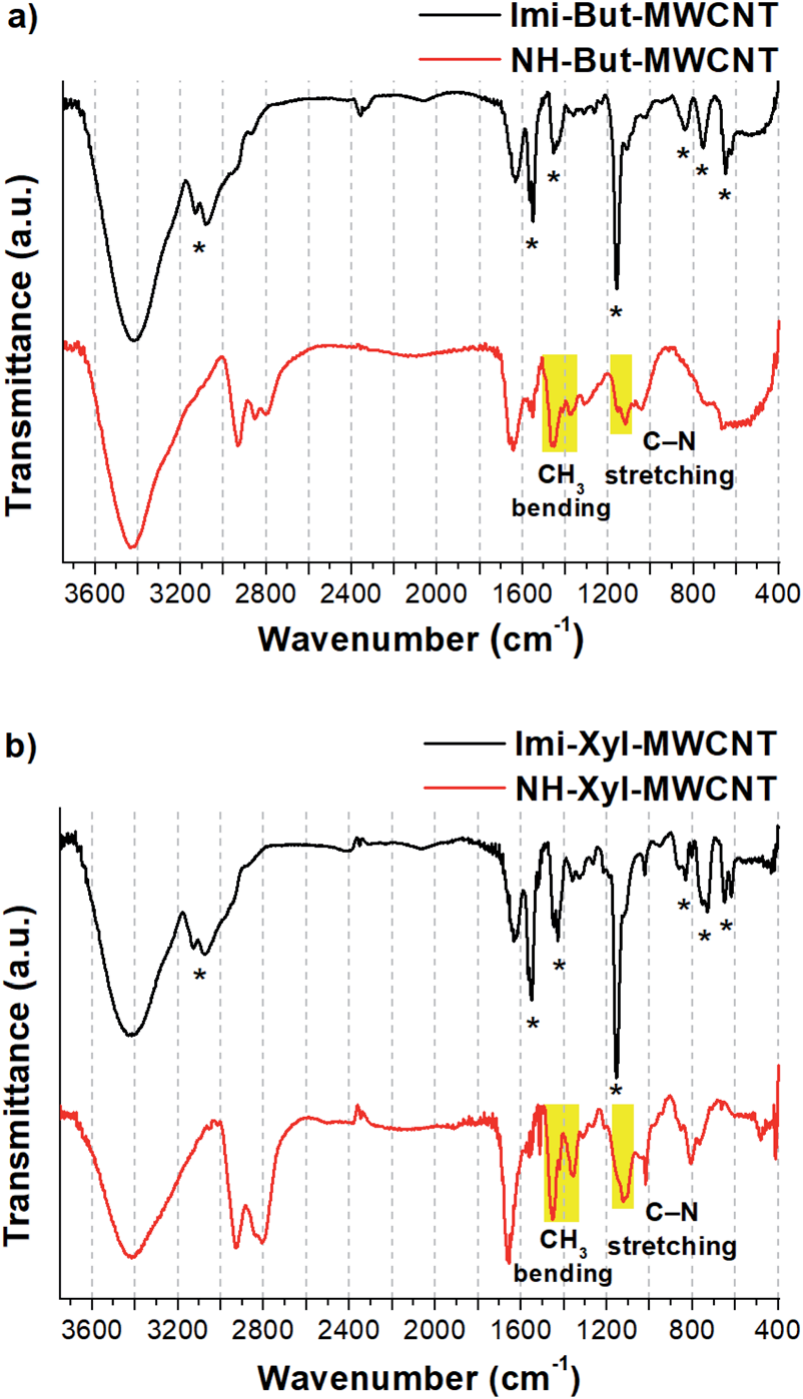
FT-IR spectra (KBr) of (a) Imi-But-MWCNT and NH-But-MWCNT and (b) Imi-Xyl-MWCNT and NH-Xyl-MWCNT. Starred bands correspond to imidazolium vibration modes (for attribution see ref. 88 and 89).

FT-IR spectroscopy (Fig. 3) confirmed the results of solid-state NMR experiments and provided further proof of the successful reduction of Imi-Xyl-MWCNT. In the IR spectrum of Imi-But-MWCNT (black line in Fig. 3a) the stretching of aromatic C–H of imidazolium moieties generated two clearly visible bands at 3132 and 3077 cm^−1^. Stretching of the aliphatic C–H was also present as shoulders below 3000 cm^−1^. The broad peak of medium intensity at 1629 cm^−1^ is ascribed to the H–O–H bending of residual water. Imidazolium ring stretching vibration modes were detected with medium and strong absorptions at 1551, 1454, and 1159 cm^−1^.^89^ The small absorption bands at 840, 754, and 649 cm^−1^ were ascribed to the ring bending modes of the imidazolium ring.^88,89^ The IR spectrum of Imi-Xyl-MWCNT (black line in Fig. 3b) resembles that of Imi-But-MWCNT with minor changes. The substitution of the butyl linker with the xylyl linker was reflected in the presence of a typical small and sharp band at 1019 cm^−1^ due to the in-plane bending vibrations of aromatic C–H, and a notably decreased intensity of the band around 3000 cm^−1^ due to the aliphatic C–H stretching modes.

The reduction process for obtaining materials NH-But-MWCNT and NH-Xyl-MWCNT led to the disappearance of all the signals ascribed to the imidazolium rings and the appearance of other absorption bands due to the increased contribution of the aliphatic moiety (red lines in Fig. 3). In the IR spectra of both reduced materials, increased signal intensity ascribed to the aliphatic C–H stretching vibration modes (3000–2800 cm^−1^ region) was detected. It is worth highlighting the appearance of signals centred at 1460 and 1364 cm^−1^ (clearly visible in Fig. 3b, red line) assigned to CH_3_ bending vibration modes. In the region 1160–1120 cm^−1^ it was possible to observe the presence of weak signals due to the stretching vibration of C–N of the secondary and tertiary amines. However, in the NH-Xyl-MWCNT IR spectrum (red line, Fig. 3b), these signals were partially overlapped with those ascribed to the in-plane bending of aromatic C–H (1123 and 1019 cm^−1^), whereas the out-of-plane bending band of aromatic C–H was detected at 808 cm^−1^.

**Fig. 4:**
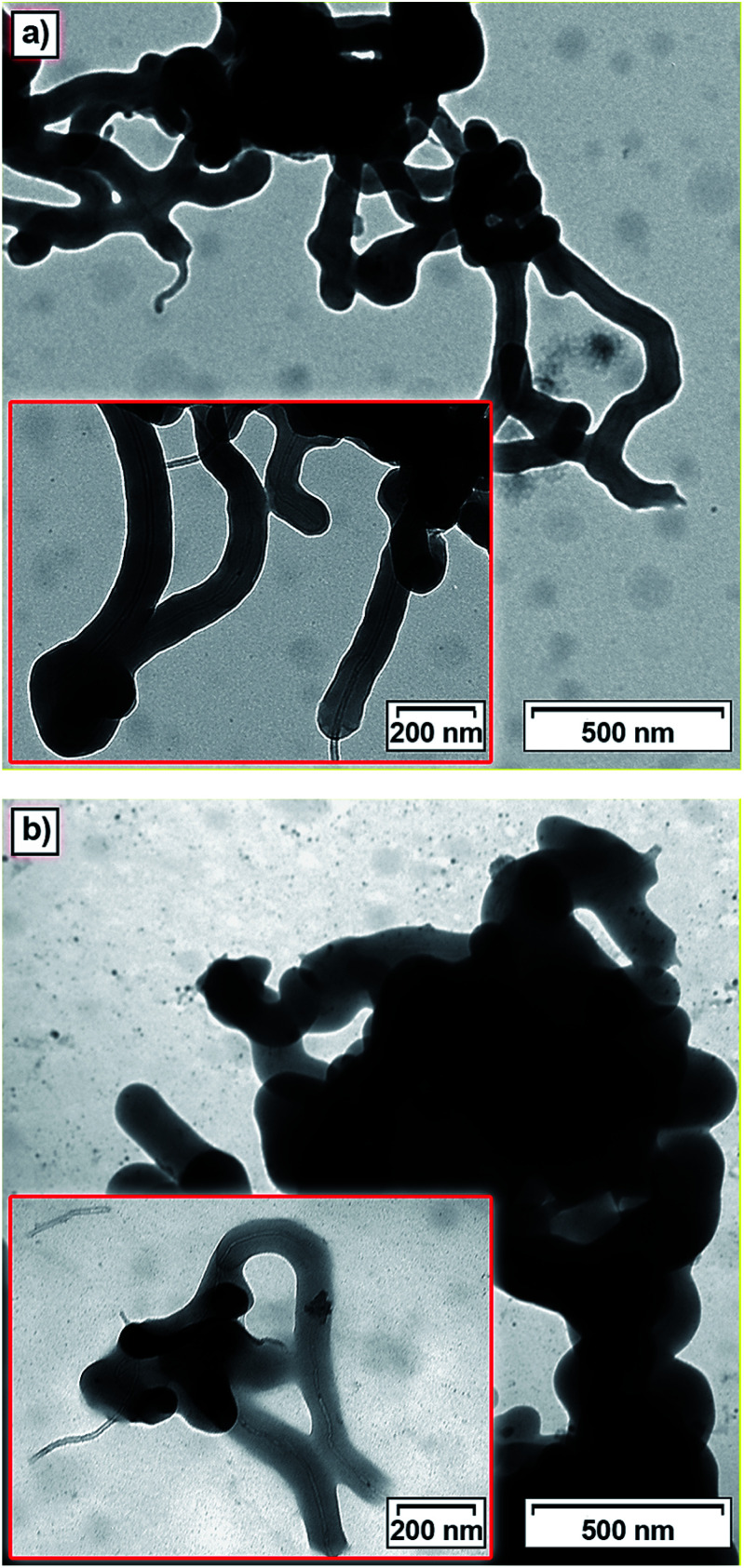
Transmission electron microscopy (TEM) images of (a) Imi-Xyl-MWCNT and (b) NH-Xyl-MWCNT.

The morphology of the prepared materials was investigated by means of transmission electron microscopy (TEM). TEM micrographs of Imi-Xyl-MWCNT and NH-Xyl-MWCNT are shown in Fig. 4 (TEM images of Imi-But-MWCNT and NH-But-MWCNT are displayed in Fig. S1†). As previously reported by us,^82^ when imidazolium salt 1a was used to functionalise MWCNTs affording a highly organised structure in which carbon nanotubes were coaxially wrapped by a polymer coating, Imi-Xyl-MWCNT also showed a similar morphology in which MWCNTs acted as a scaffold for the polymerisation of the highly cross-linked imidazolium network that perfectly covered the whole surface of nanotubes creating a cylindrical coating (Fig. 4a). TEM images of NH-Xyl-MWCNT are displayed in Fig. 4b. The comparison of these micrographs with those of Imi-Xyl-MWCNT allows us to make some conclusions. As a matter of fact, if on the one hand TEM images of NH-Xyl-MWCNT showed that reduction of material Imi-Xyl-MWCNT involved no remarkable changes in the morphology of the material, on the other hand they provided proof that the reduction process causes no relevant alteration of the polymeric network around the nanotubes.

**Fig. 5:**
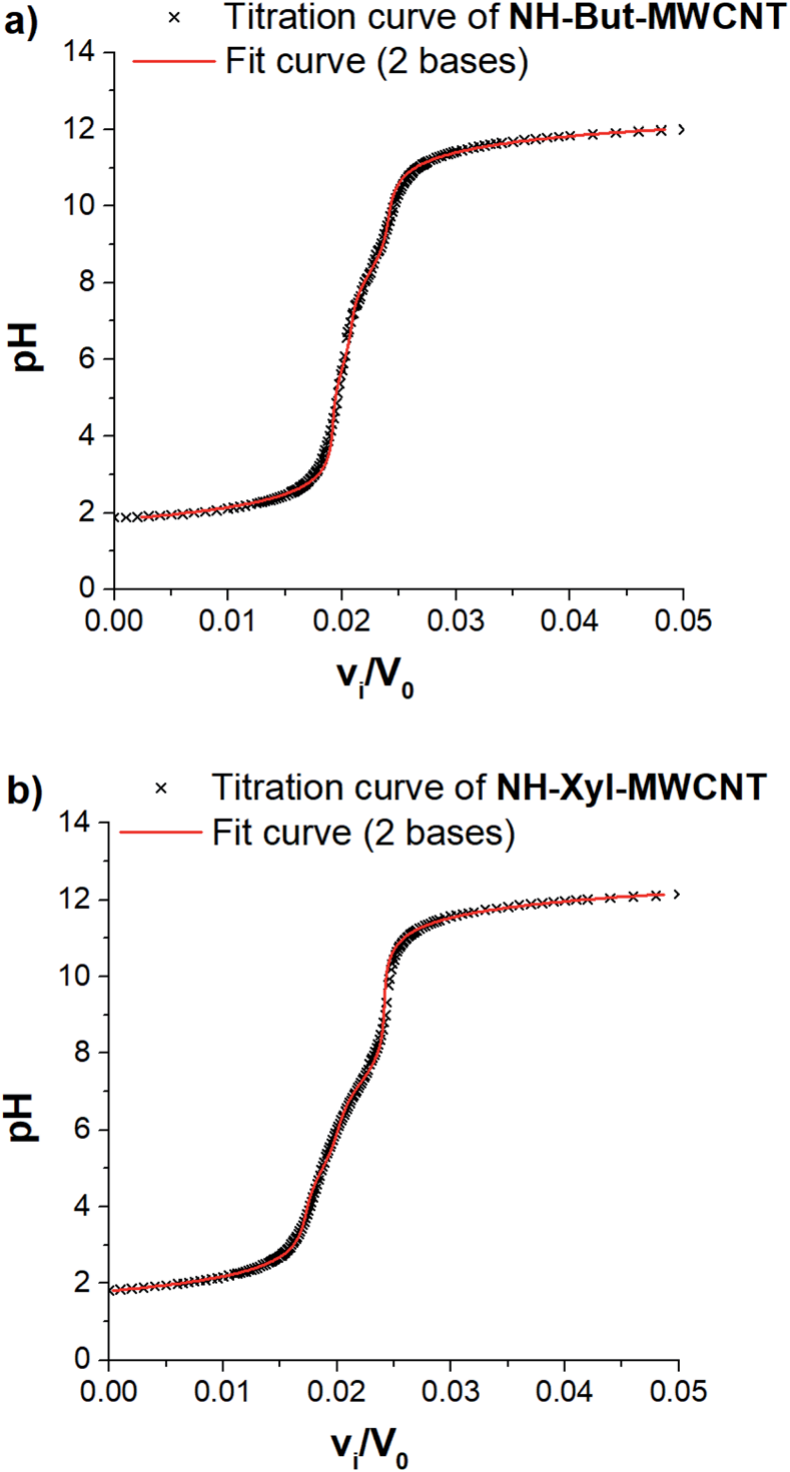
Potentiometric titration of (a) NH-But-MWCNT and (b) NH-Xyl-MWCNT.

Taking into account the highly cross-linked nature of materials NH-But-MWCNT and NH-Xyl-MWCNT, it is reasonable to hypothesize that the amine groups present in the materials might not equally perform their function because some of them could be hardly accessible. This is an aspect of general importance, and a reliable estimation of the active amino groups represents a powerful tool to assess the real amino group contents of the studied materials. A simple way to quantitatively assess the number of accessible amino groups is the evaluation of their proton acceptor ability estimated by an acid–base potentiometric titration. More in detail, the aqueous suspensions of reduced materials in the presence of an excess of a strong acid (HCl) were titrated with a standard 1 M NaOH solution. According to a reported procedure,^90,91^ the system is modelled as a mixture of independent monoprotic weak bases; then the experimental titration curves (Fig. 5) are subjected to a regression analysis using the relevant mathematical expression (eqn (1)) obtained analytically:1
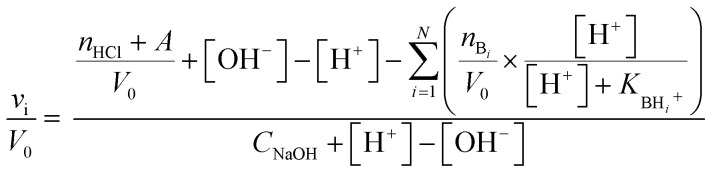
where *v*_i_ is the added volume of the titrant solution, *V*_0_ the initial volume of the suspension, *n*_HCl_ the moles of HCl, *C*_NaOH_ the concentration of NaOH solution, and *A* a correction parameter inserted to take into account the possible presence of strong acids or bases in trace amounts coming from the workup process. Eqn (1) can be trivially transformed in such a way to have pH as the sole independent variable by taking [H^+^] = 10^−pH^, and [OH^−^] = 10^−p*K*_w_^/10^−pH^.

In our case, data analysis led to the conclusion that a sum of two independent weak bases is the best compromise to suitably model the behaviour of our systems. The relevant fitting parameters are collected in Table 1.

**Table 1:** Calculated parameters obtained by the regression analysis of the experimental titration curves of NH-But-MWCNT and NH-Xyl-MWCNT

Material	*A* (mmol)	*n*_B_1__/g (mmol g^−1^)	*n*_B_2__/g (mmol g^−1^)	p*K*_BH_1_^+^_	p*K*_BH_2_^+^_	Amino group content (mmol g^−1^)
NH-But-MWCNT	−0.018 ± 0.002	1.6 ± 0.2	3.2 ± 0.2	5.78 ± 0.29	8.28 ± 0.11	4.8 ± 0.4
NH-Xyl-MWCNT	−0.017 ± 0.001	2.2 ± 0.1	3.2 ± 0.1	4.98 ± 0.08	7.24 ± 0.05	5.4 ± 0.2

It is very important to note that the reported p*K*_BH^+^_ values represent only apparent values related to the two independent weak bases assumed for the analytical derivation of eqn (1). However, some remarks can be made after an important premise. In the case of the materials studied and polyamines in general,^92,93^ protonation of each amino group is influenced (lowering its basicity) by the presence of other previously protonated (charged) amino groups directly linked on the backbone. Furthermore, the highly cross-linked nature of materials NH-But-MWCNT and NH-Xyl-MWCNT will tend to increase the charge density after the protonation of amino groups, further reducing the basicity of non-protonated ones because of the charge repulsion. Now, if one would try to rationalise the p*K*_BH^+^_ values obtained, they could represent a measure of the protonation affinity of amino groups located at the external surface (p*K*_BH_2_^+^_) and in the inner regions (p*K*_BH_1_^+^_) of the polymer coating. A similar behaviour has been studied for inner and outer shells in poly(propylene imine) dendrimers.^94^

The comparison of the amino group contents reported in Table 1 with those obtained by TGA showed that only 38% and 53% of the total amount of amino groups present in the cross-linked polymers were revealed by potentiometric titration for NH-But-MWCNT and NH-Xyl-MWCNT, respectively. This finding is consistent with the behaviour reported for some nanosponge materials,^95^ which was explained with the presence in the materials of highly hydrophobic regions hardly accessible to the aqueous medium. It is worth noting that both materials showed similar results concerning the amino group content on their external surface, whereas material NH-Xyl-MWCNT exhibited a higher content of amino groups in its inner surface than NH-But-MWCNT (Table 1). The largest differences could be noticed by analysing the p*K*_BH^+^_ values, since material NH-Xyl-MWCNT showed fewer basic amino groups. All these results could be explained by taking into account the different nature of the linker in the two materials. In fact, in the case of material NH-Xyl-MWCNT, if on the one hand the xylyl linker could provide higher rigidity to the polymeric network improving the spacing between protonated amino groups and reducing the charge density, on the other hand, it causes the basicity reduction of amino groups as evidenced by the lower p*K*_BH^+^_ values.

**Fig. 6:**
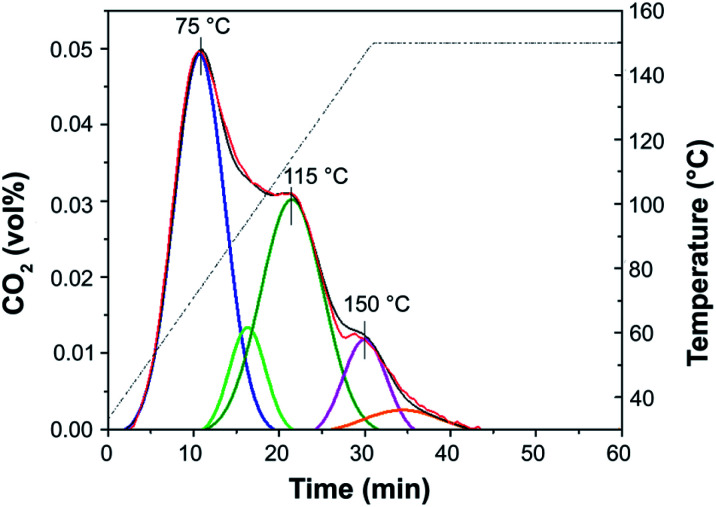
CO_2_-TPD of NH-But-MWCNT.

Temperature-programmed desorption of carbon dioxide (CO_2_-TPD) was used to determine the basic site distribution. Deconvolution of the CO_2_-TPD profile of NH-But-MWCNT reported in Fig. 6 indicates the presence of basic sites having different nature as demonstrated by the desorption of CO_2_ at different temperature. This behaviour could be related both to the presence of tertiary/secondary amines and exposed and bulky layers as in the case of polyethylenimine on SBA-15.^96^ In addition, CO_2_-TPD carried out on Imi-But-MWCNT showed no CO_2_ desorption, indicating the completely different nature of the unreduced and reduced materials.

Among C–C bond forming reactions, Knoevenagel condensation, Michael addition, and Henry reaction play a pivotal role in the synthesis of pharmaceuticals or fine chemicals.^97,98^ The possibility to catalyse these types of reactions by the use of heterogeneous base catalysts prompted us to investigate the catalytic activity of the prepared materials in such chemical transformations.

Materials NH-But-MWCNT and NH-Xyl-MWCNT were initially tested as heterogeneous base catalysts in the Knoevenagel condensation. The reaction between 4-bromobenzaldehyde and ethyl cyanoacetate was chosen as a model reaction to test the catalytic activity of both materials. Preliminary results, when carrying out the reaction in ethanol at 30 °C for 1 h and using a catalytic loading of 1 mol%, showed moderate conversions into the desired product with no significant differences between the two catalysts (Table 2, entries 1a and 2a). The increase of the catalytic loading up to 2 mol% allowed reaching higher conversions (Table 2, entries 1b and 2b).

**Table 2:** Knoevenagel condensations between aromatic aldehydes and ethyl cyanoacetate^a^

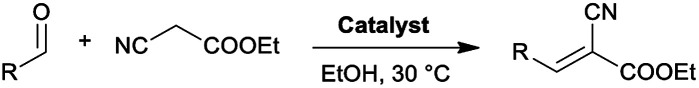					
Entry	Catalyst	R	Catal. loading (mol%)	*t* (h)	Conv.^b^ (%)
1a	NH-But-MWCNT	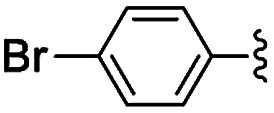	1	1	38
1b			2	1	97
2a	NH-Xyl-MWCNT		1	1	44
2b			2	1	80
3	NH-But-MWCNT	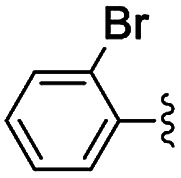	2	18	97
4	NH-But-MWCNT	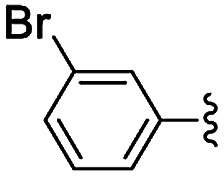	2	2	>99
5	NH-But-MWCNT	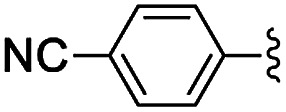	2	1	98
6	NH-But-MWCNT	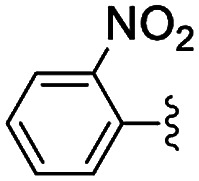	2	2	96
7	NH-But-MWCNT	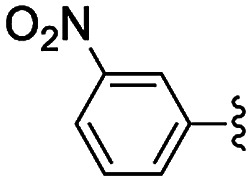	2	0.5	>99
8	NH-But-MWCNT	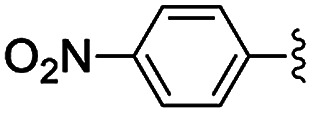	2	0.5	>99
9	NH-But-MWCNT	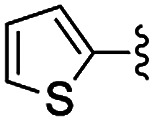	2	6	89
10	NH-But-MWCNT	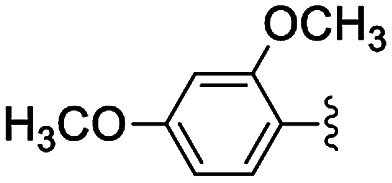	2	2	88
11	NH-But-MWCNT	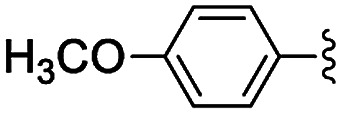	2	18	89

a Reaction conditions: 1.27 mmol of 4-bromobenzaldehyde, 1.27 mmol of ethyl cyanoacetate, NH-But-MWCNT or NH-Xyl-MWCNT (1 mol% or 2 mol%), 190 μL of ethanol, 30 °C, 1 h (for entries 1a–2b); 1 mmol of aldehyde, 1 mmol of ethyl cyanoacetate, 1.58 mg of NH-But-MWCNT (2 mol%), 150 μL of ethanol, 30 °C (for entries 3–11).

b Determined by ^1^H NMR.

In the light of the higher catalytic activity, material NH-But-MWCNT was chosen to carry out other catalytic tests by reacting different aldehydes with ethyl cyanoacetate using a 2 mol% catalytic loading (Table 2, entries 3–11). In general, aromatic aldehydes with electron-withdrawing substituents (Table 2, entries 3–8) gave rise to excellent conversions in short times, with the exception of 2-bromobenzaldehyde which required more time (Table 2, entry 3). Both a heteroaromatic aldehyde and aromatic aldehydes with electron-donating groups were tested (Table 2, entries 9–11). Good conversions were achieved even though it was necessary to increase the reaction time in the case of 2-thiophenecarboxaldehyde and 4-methoxybenzaldehyde (Table 2, entries 9 and 11).

**Fig. 7:**
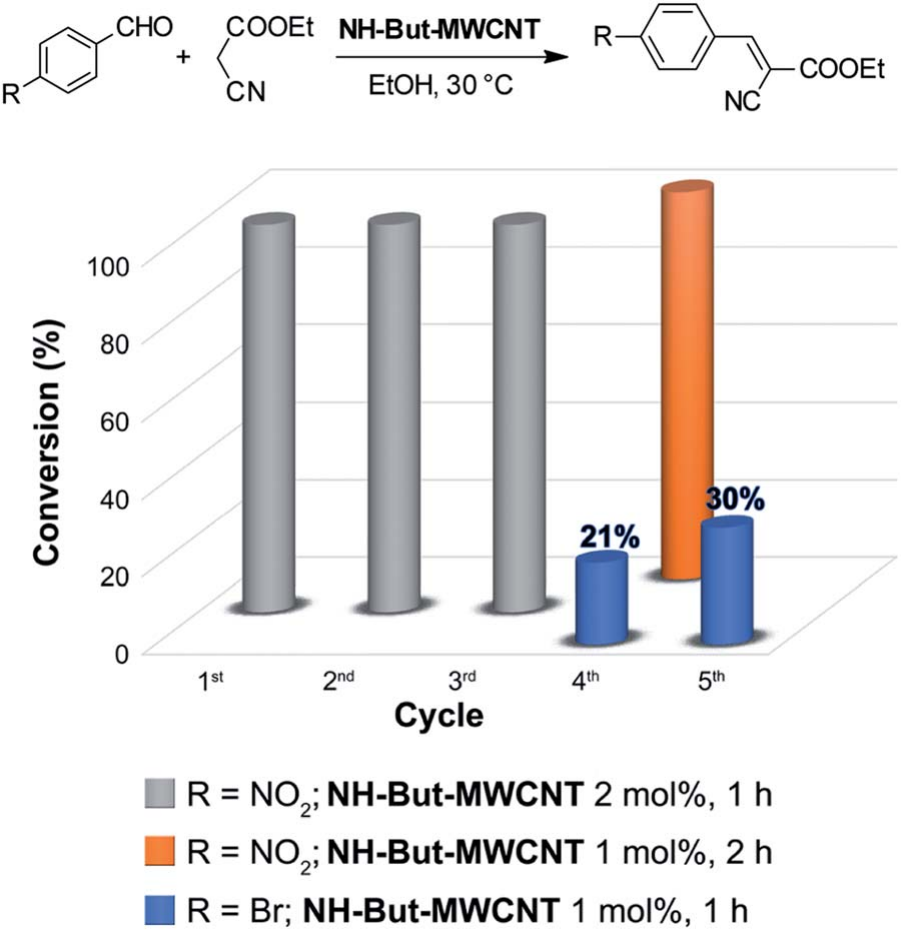
Recycling experiments of the Knoevenagel reaction.

The reaction between 4-nitrobenzaldehyde and ethyl cyanoacetate, performed in ethanol at 30 °C for 1 h with a catalyst loading of 2 mol%, was chosen to assess the recyclability of material NH-But-MWCNT (Fig. 7). NH-But-MWCNT was easily recovered by centrifugation and allowed a complete conversion of 4-nitrobenzaldehyde into the desired product with no loss of catalytic activity for three consecutive cycles. After the third cycle, the recovered catalyst was split into two portions. One portion was used to carry out the same reaction with a lower catalyst loading of 1 mol%, whereas the second portion was employed for the catalysis of the Knoevenagel condensation between 4-bromobenzaldheyde and ethyl cyanoacetate using the same catalyst loading of 1 mol%. The results, reported in Fig. 7, showed that a complete conversion was achieved in the first case, despite the lower amount of catalyst employed. On the other hand, the use of NH-But-MWCNT at 1 mol% for the conversion of 4-bromobenzaldehyde into the corresponding Knoevenagel adduct gave rise to a conversion value of 21%, lower than the one obtained with the fresh catalyst (compare Fig. 7 and Table 2, entry 1a). An acid treatment of the recovered catalyst NH-But-MWCNT followed by a subsequent basic washing was performed in order to verify if the catalytic activity could be restored. The results revealed that there was a partial recovery of the starting activity achieving a conversion value of 30% (Fig. 7), leading us to hypothesise a plausible catalyst deactivation mechanism that involves the unreacted ethyl cyanoacetate.^99^ The active basic sites could react by an acid–base reaction with ethyl cyanoacetate, which could eventually block the amino groups by an electrostatic interaction. In this scenario, the use of less reactive substrates, such as 4-bromobenzaldehyde, whose reactivity toward the nucleophilic addition to the carbonyl moiety could be strongly affected by the possible formation of the iminium intermediate,^100^ resulted in a decreased catalytic activity of the recycled catalyst (compare the 4th cycle in Fig. 7 and Table 2, entry 1a). However, the acid and basic washings of the spent catalyst seemed to be sufficient to regenerate the basic active sites, which became available for the next cycle restoring part of the initial catalytic activity. On the other hand, when more reactive 4-nitrobenzaldehyde was used, no decrease of catalytic activity was revealed.

Material NH-But-MWCNT was also employed as the catalyst of a sequential one-pot process, namely the consecutive Knoevenagel and Michael reactions. The preliminary results are reported in Table 3 and further investigations are being undertaken by our research group. Both 4-bromo- and 4-nitrobenzaldehyde were first subjected to the Knoevenagel reaction with ethyl cyanoacetate giving rise to the complete conversion into the corresponding intermediates. The subsequent addition of nitromethane to the same reaction vessel without any change in the reaction conditions led to the complete conversions into the Michael adducts. Material NH-But-MWCNT was tested in another base catalysed C–C bond forming reaction, namely the Henry reaction. The reaction between 4-bromobenzaldehyde and nitromethane was chosen to find the optimal reaction conditions (Table 4). The first attempts were addressed towards the use of solvent-free conditions (Table 4, entries 1–2b). The use of NH-But-MWCNT, with a loading of 2.5 mol% at 50 °C and a reaction time of 18 h, gave rise to moderate conversion of the starting reagents and good selectivity towards the desired product (Table 4, entry 1). Increasing the loading up to 5 mol% allowed similar results to be reached with slightly improved conversion in only 6 h (Table 4, entry 2a). However, a greater reaction time of 24 h, if on the one hand improved the conversion value up to 88%, on the other hand led to a reduction of selectivity (Table 4, entry 2b). Modification of the reaction conditions, both decreasing the amount of nitromethane (5 equiv.) and using ethanol as the solvent without affecting the other parameters, led to no improvement in conversion and selectivity (Table 4, entry 3). The use of 10 equiv. of nitromethane and raising the temperature to 80 °C allowed the same results to be reached in less time (Table 4, entry 4a), but the further progress of the reaction up to 24 h caused a marked decrease of the selectivity (Table 4, entry 4b). The decrease in selectivity was ascribed to the detrimental effect of the temperature which could favour the dehydration of the Henry adduct that undergoes subsequent Michael addition of a second equivalent of nitromethane. Therefore, the same reaction conditions used in the case of entry 4b, decreasing the temperature down to room temperature, were adopted with the aim of minimizing the formation of the by-product (Table 4, entry 5). Good conversion and excellent selectivity were achieved and these optimized conditions were chosen to explore the substrate scope of material NH-But-MWCNT by reacting different aldehydes with nitromethane (Table 5).

**Table 3:** Sequential one-pot Knoevenagel/Michael reaction

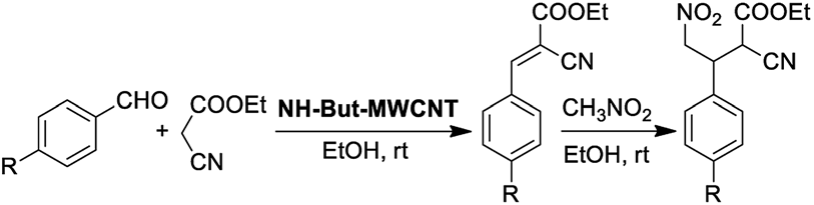				
Entry	R	*t* (h) step I	*t* (h) step II	Total conv.^a^ (%)
1^b^	4-Br	2	20^c^	>99
2^d^	4-NO_2_	1	1.5	>99

a Determined by ^1^H NMR.

b Reaction conditions: 1 mmol of 4-bromo-benzaldehyde, 1 mmol of ethyl cyanoacetate, 10 mmol of CH_3_NO_2_, 1.58 mg of NH-But-MWCNT (2 mol%), 150 μL of ethanol, rt.

c Reaction time not optimized.

d Reaction conditions: 2.5 mmol of 4-bromobenzaldehyde, 2.5 mmol of ethyl cyanoacetate, 25 mmol of CH_3_NO_2_, 3.94 mg of NH-But-MWCNT (2 mol%), 375 μL of ethanol, rt.

**Table 4:** Screening of the reaction conditions for the Henry reaction^a^

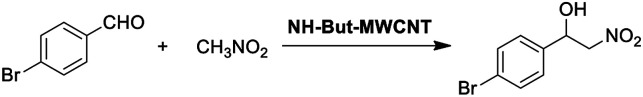						
Entry	Catal. loading (mol%)	*T* (°C)	Equiv. CH_3_NO_2_	Solvent	*t* (h)	Conv. [selectivity]^b^ (%)
1	2.5	50	37	—	18	66 [91]
2a	5	50	37	—	6	76 [94]
2b	5	50	37	—	24	88 [87]
3	5	50	5	EtOH	24	78 [89]
4a	5	80	10	EtOH	7	80 [84]
4b	5	80	10	EtOH	24	85 [40]
5	5	Rt	10	EtOH	24	87 [97]

a Reaction conditions: 0.245 mmol of 4-bromobenzaldehyde, CH_3_NO_2_, NH-But-MWCNT, (345 μL of ethanol for entries 3–5).

b Determined by ^1^H NMR.

**Table 5:** Henry reactions between aromatic aldehydes and nitromethane^a^

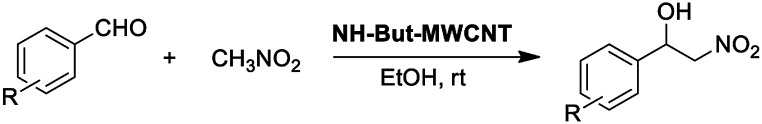			
Entry	R	*t* (h)	Conv. [selectivity]^b^ (%)
1	3-Br	24	76 [97]
2	2-Br	24	41 [99]
3	4-NO_2_	24	98 [99]
4	3-NO_2_	24	97 [98]
5	2-NO_2_	24	80 [99]
6	4-CN	24	87 [96]
7	4-OMe	24	<5
8	2,4-OMe	24	32 [64]

a Reaction conditions: 0.245 mmol of aldehyde, 10 equiv. of CH_3_NO_2_, 1 mg of NH-But-MWCNT (5 mol%), 345 μL of ethanol, rt.

b Determined by ^1^H NMR.

Selectivity towards the desired products was excellent with all the substituted aromatic aldehydes with electron-withdrawing groups (Table 5, entries 1–6). Conversions were higher than 76% with the exception of the reaction involving 2-bromobenzaldehyde which was converted at 41% (Table 5, entry 2). The reaction with electron-rich aldehydes led to poor conversion in the case of 4-methoxybenzaldehyde (Table 5, entry 7) and low conversion and selectivity when 2,4-dimethoxybenaldehyde was reacted with nitromethane (Table 5, entry 8).

## Conclusions

In summary, two materials based on MWCNTs functionalised with highly cross-linked polyamines were prepared by means of radical polymerisation of two bis-vinylimidazolium salts onto the carbonaceous support followed by their reduction with sodium borohydride and thoroughly characterized by means of spectroscopic and analytic techniques. The reduction step was confirmed by different characterisation techniques, and a high degree of amino-functionalisation was achieved. Furthermore, potentiometric titration was used to estimate the amount of accessible basic sites. The herein presented synthetic route represents an efficient and easy way to get access to secondary and tertiary amine-functionalised materials avoiding any pre-treatment of pristine MWCNTs. The high amino group content makes the prepared materials intriguing for a wide range of applications. Herein, some C–C bond forming reactions such as Knoevenagel condensation, Michael addition, and Henry reaction were chosen to assess their function as heterogeneous base catalysts.

## Conflicts of interest

There are no conflicts to declare.

## Supplementary Material

NA-002-D0NA00291G-s001
