# Supplementary material for: Straightforward preparation of highly loaded MWCNT–polyamine hybrids and their application in catalysis
Source: Nanoscale Adv. 2020 Jul 14;2(9):4199–211. doi: 10.1039/d0na00291g (PMC9417923; doi:10.1039/d0na00291g)
Supplement: NA-002-D0NA00291G-s001 [file NA-002-D0NA00291G-s001.pdf]

Electronic Supplementary Material (ESI) for Nanoscale Advances.
This journal is © The Royal Society of Chemistry 2020

Supporting Information

Straightforward Preparation of Highly Loaded MWCNT-Polyamine Hybrids and their Application in Catalysis

Vincenzo Campisciano,[a] Rene Burger,[a,b] Carla Calabrese,[a] Leonarda Francesca Liotta,[c] Paolo Lo Meo,[a] Michelangelo Gruttadauria*[a] and Francesco Giacalone*[a]

a Department of Biological, Chemical and Pharmaceutical Sciences and Technologies, University of Palermo, Viale delle Scienze, Ed. 17, 90128 Palermo, Italy. E-mail: michelangelo.gruttadauria@unipa.it ; francesco.giacalone@unipa.it

b Department of Natural Sciences, Bonn-Rhein-Sieg University of Applied Sciences, von-Liebig-Strasse 20, D-53359 Rheinbach, Germany.

c Istituto per lo Studio dei Materiali Nanostrutturati ISMN-CNR, via Ugo La Malfa, 153, 90146 Palermo (Italy).

Table of Contents:

TEM images of **Imi-But-MWCNT** and **NH-But-MWCNT** **S2**

^{1}H NMR spectra of products reported in **Table 2** **S3**

^{1}H NMR spectra of products reported in **Table 3** **S8**

^{1}H NMR spectra of product reported in **Table 4** **S9**

^{1}H NMR spectra of products reported in **Table 5** **S9**

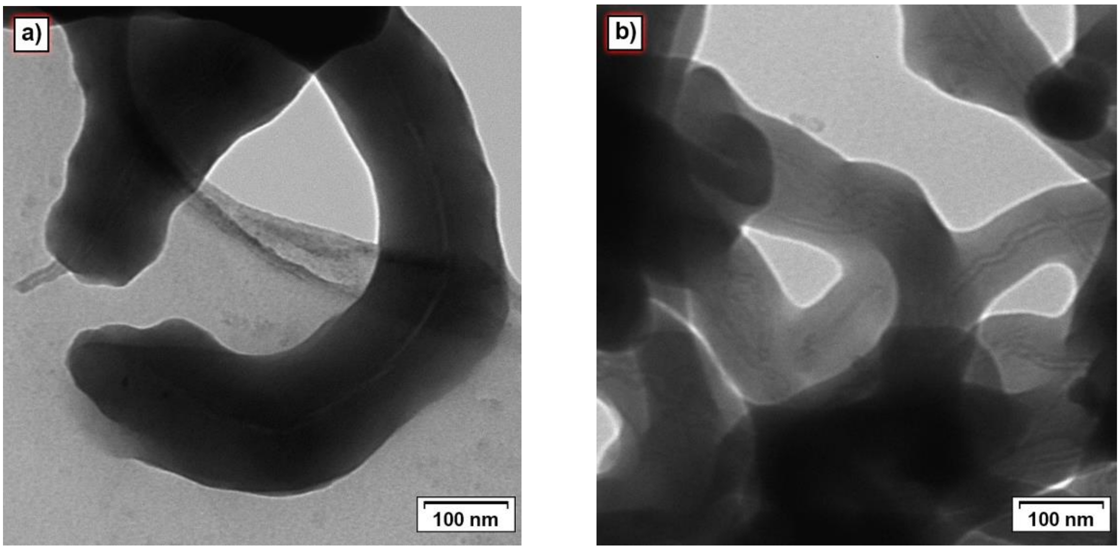

Figure S1. TEM images of a) **Imi-But-MWCNT** and b) **NH-But-MWCNT**.

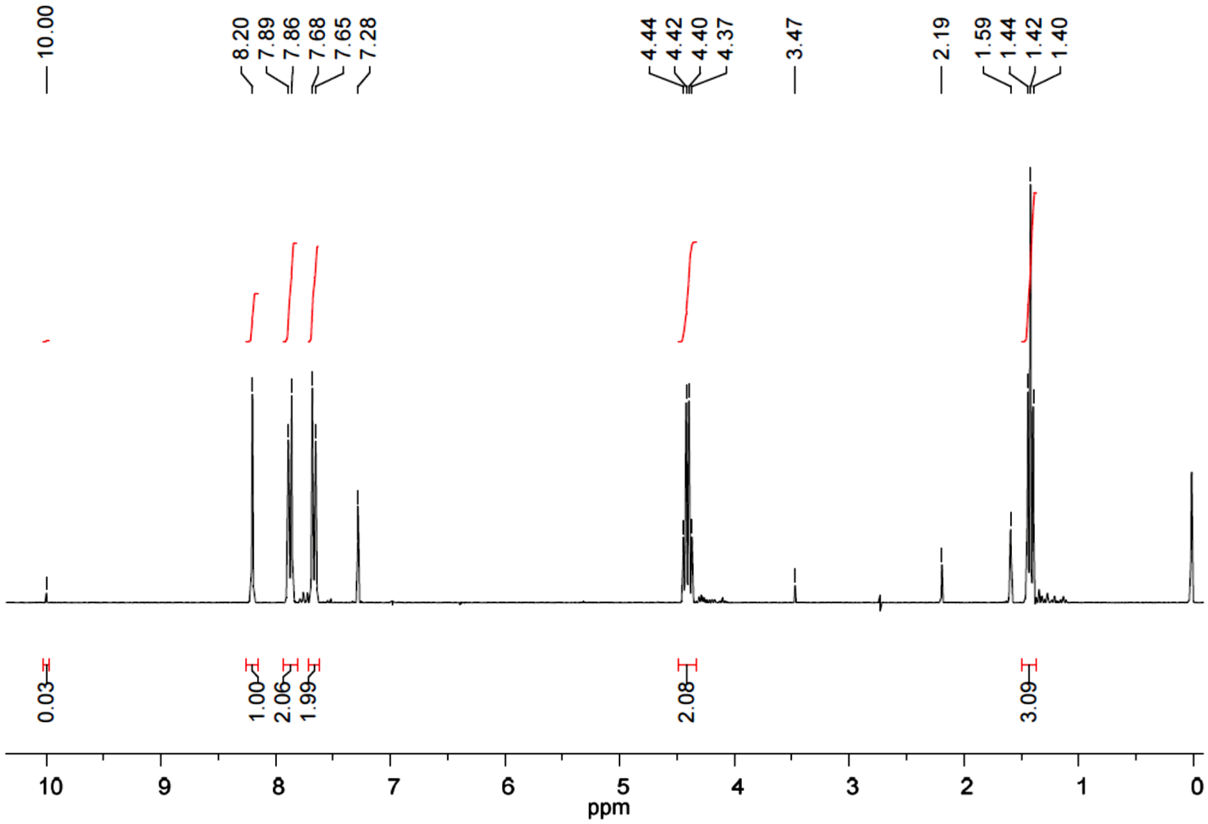

^{1}H NMR (300 MHz, $CDCl_3$) **Table 2**, **Entry 1b**.

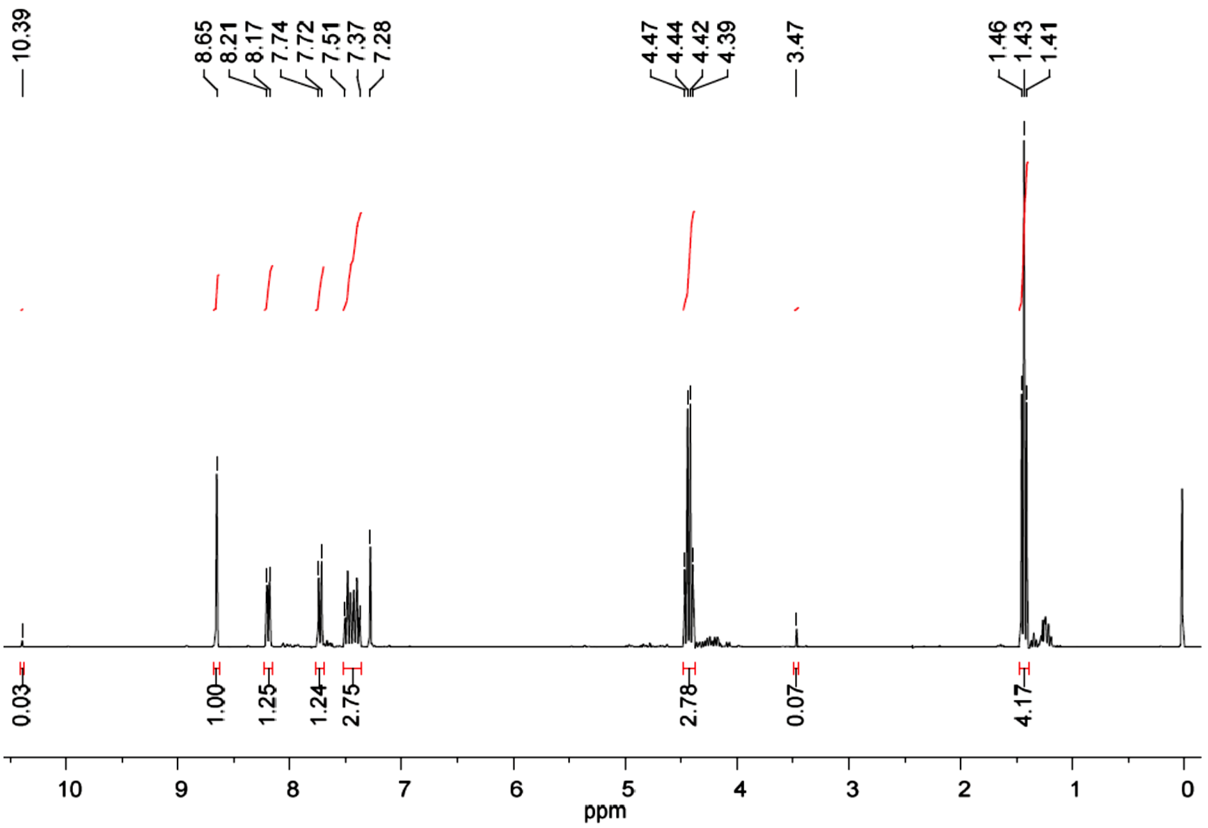

^{1}H NMR (300 MHz, $CDCl_3$) **Table 2**, **Entry 3**.

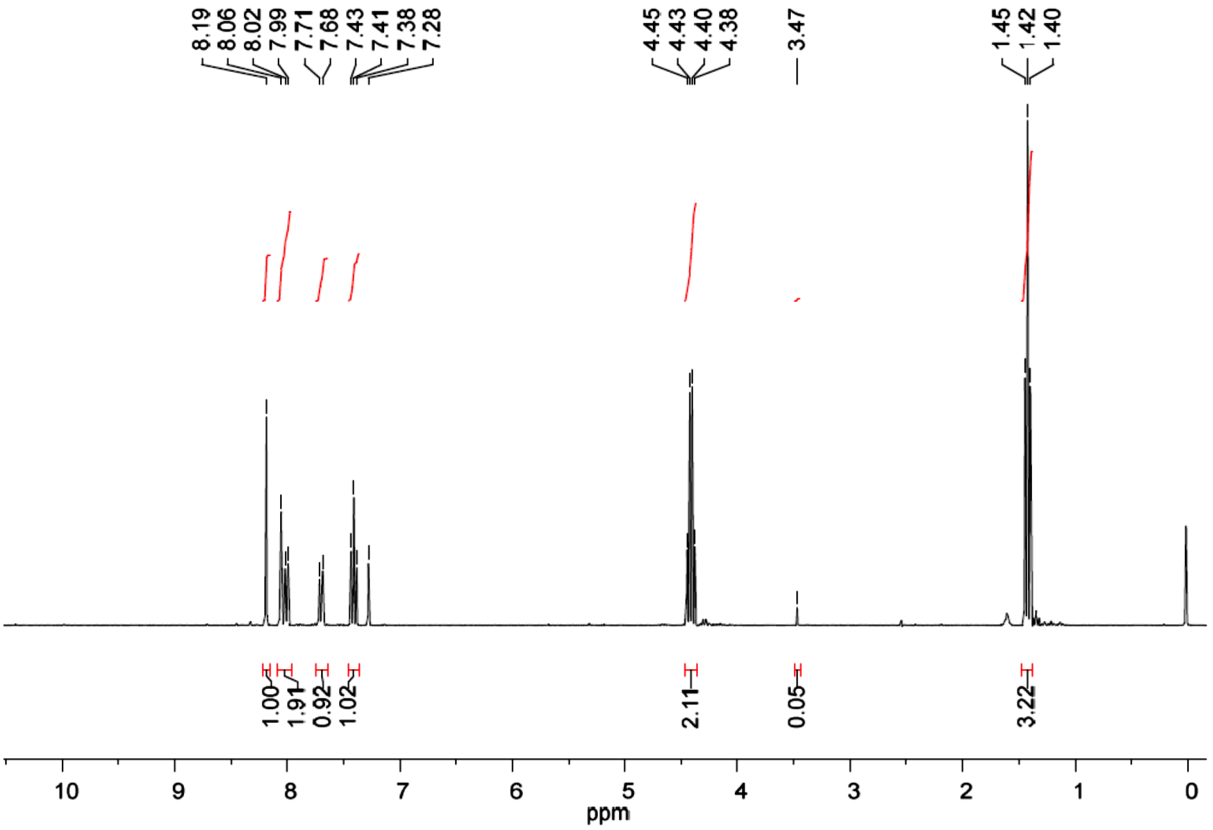

^{1}H NMR (300 MHz, $CDCl_3$) **Table 2**, **Entry 4**.

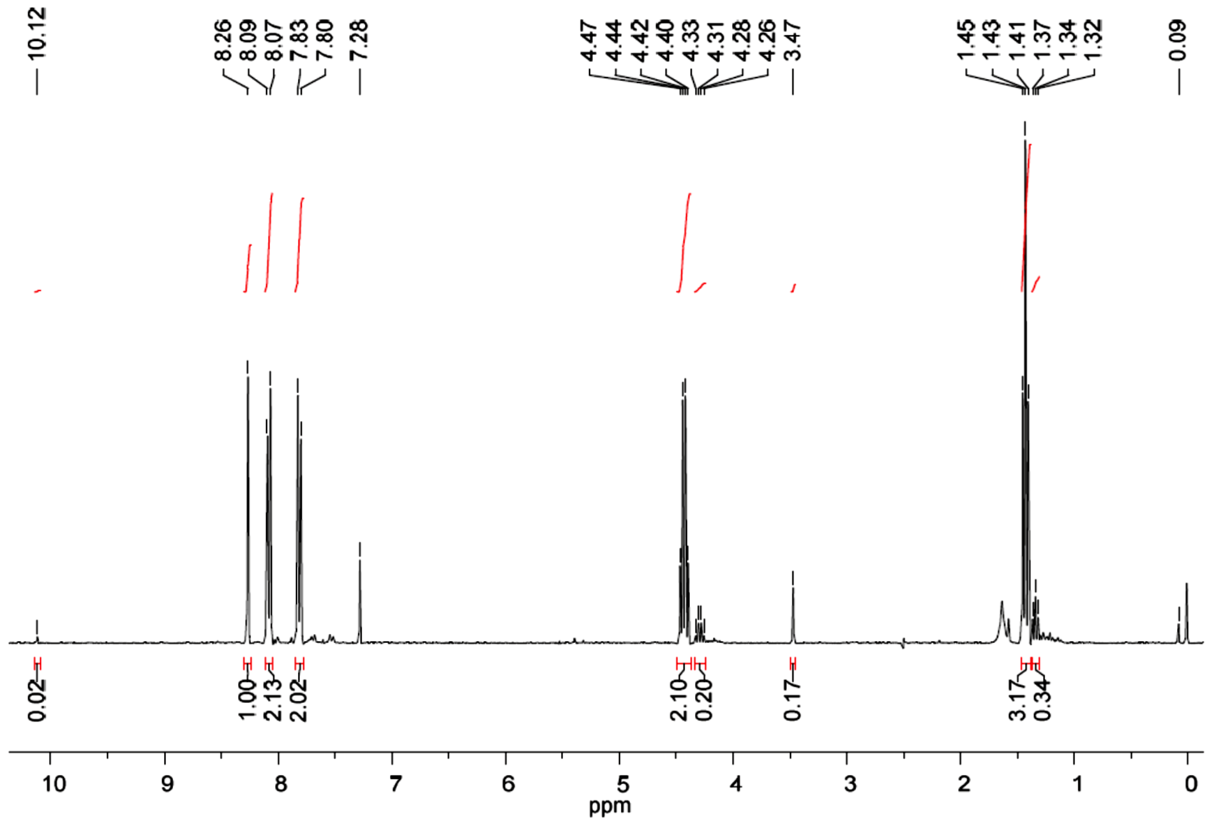

^{1}H NMR (300 MHz, $CDCl_3$) **Table 2**, **Entry 5**.

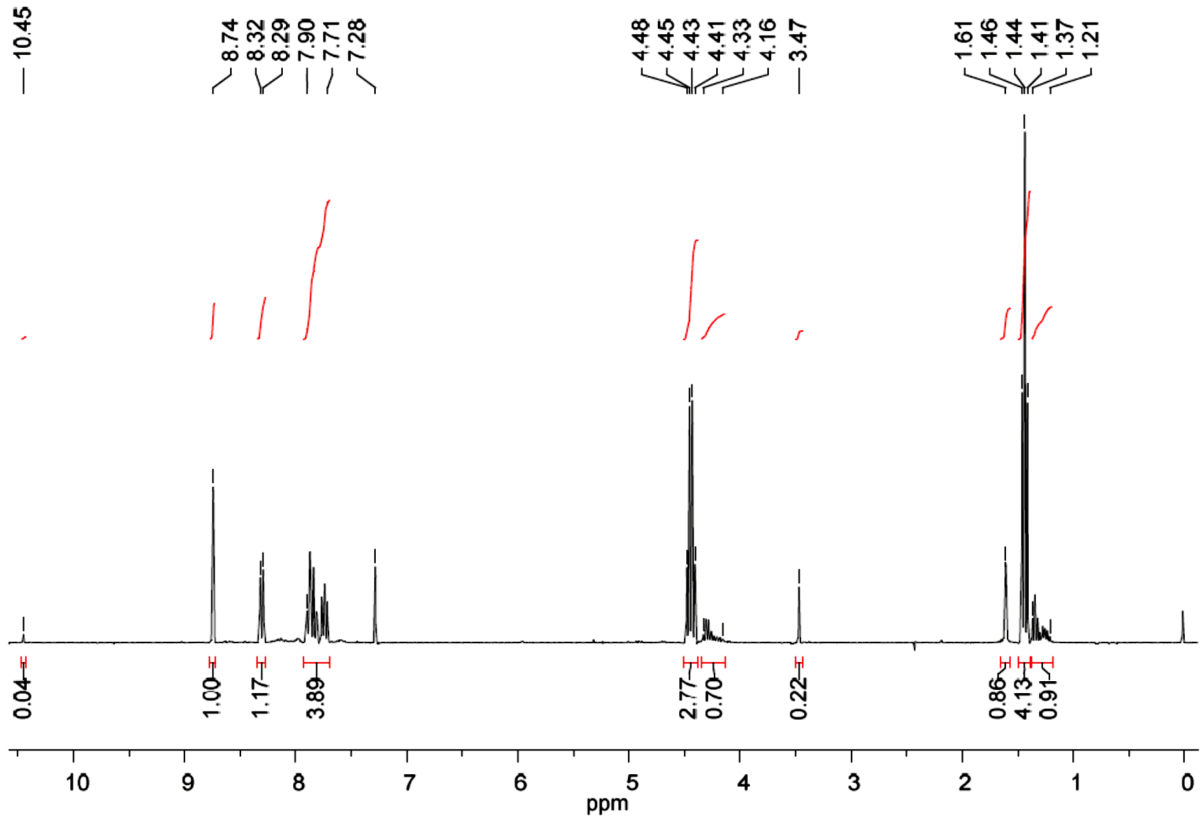

^{1}H NMR (300 MHz, $CDCl_3$) **Table 2**, **Entry 6**.

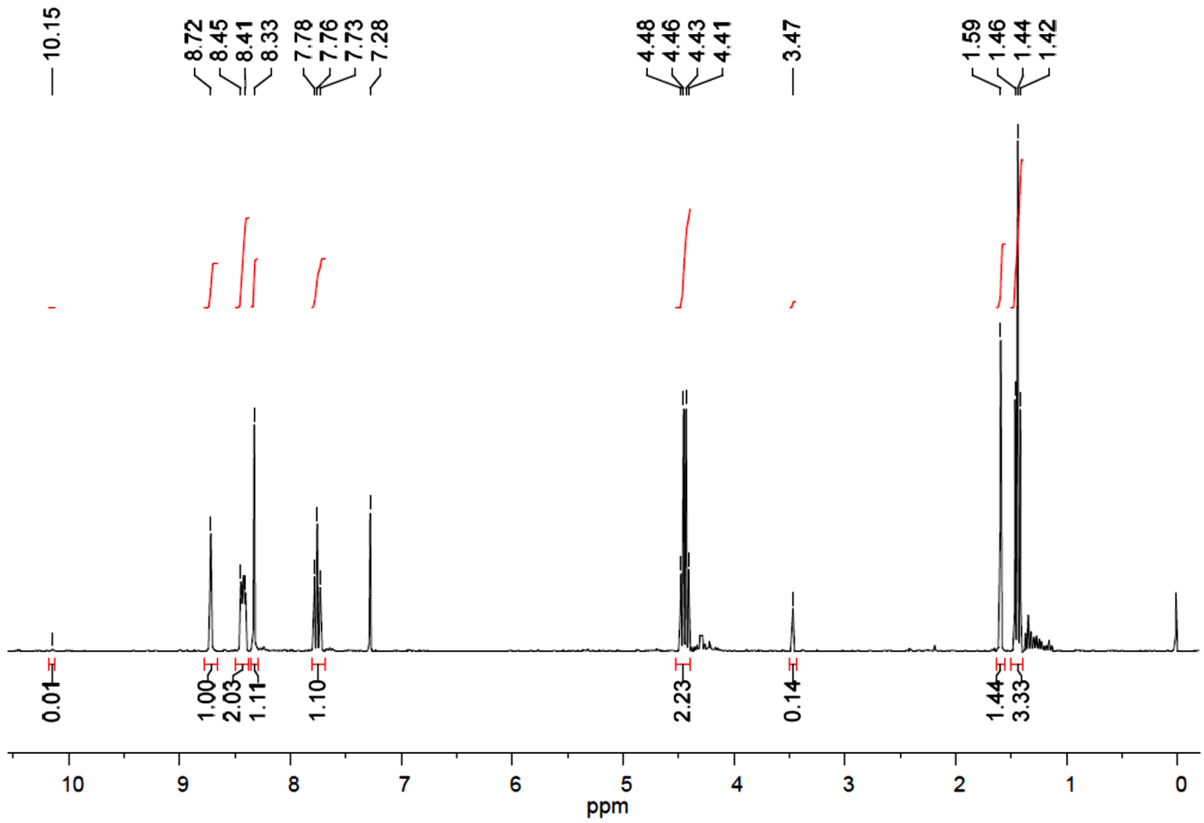

^{1}H NMR (300 MHz, $CDCl_3$) **Table 2**, **Entry 7**.

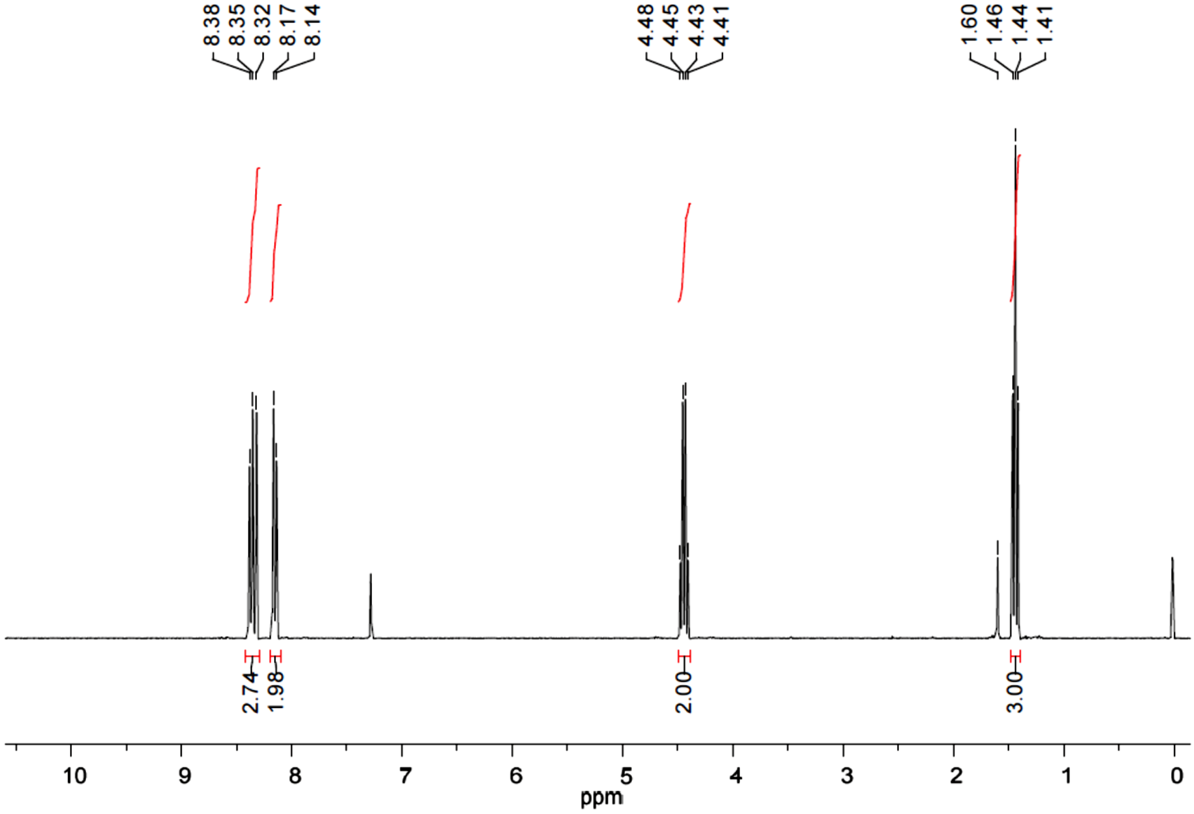

^{1}H NMR (300 MHz, $CDCl_3$) **Table 2**, **Entry 8**.

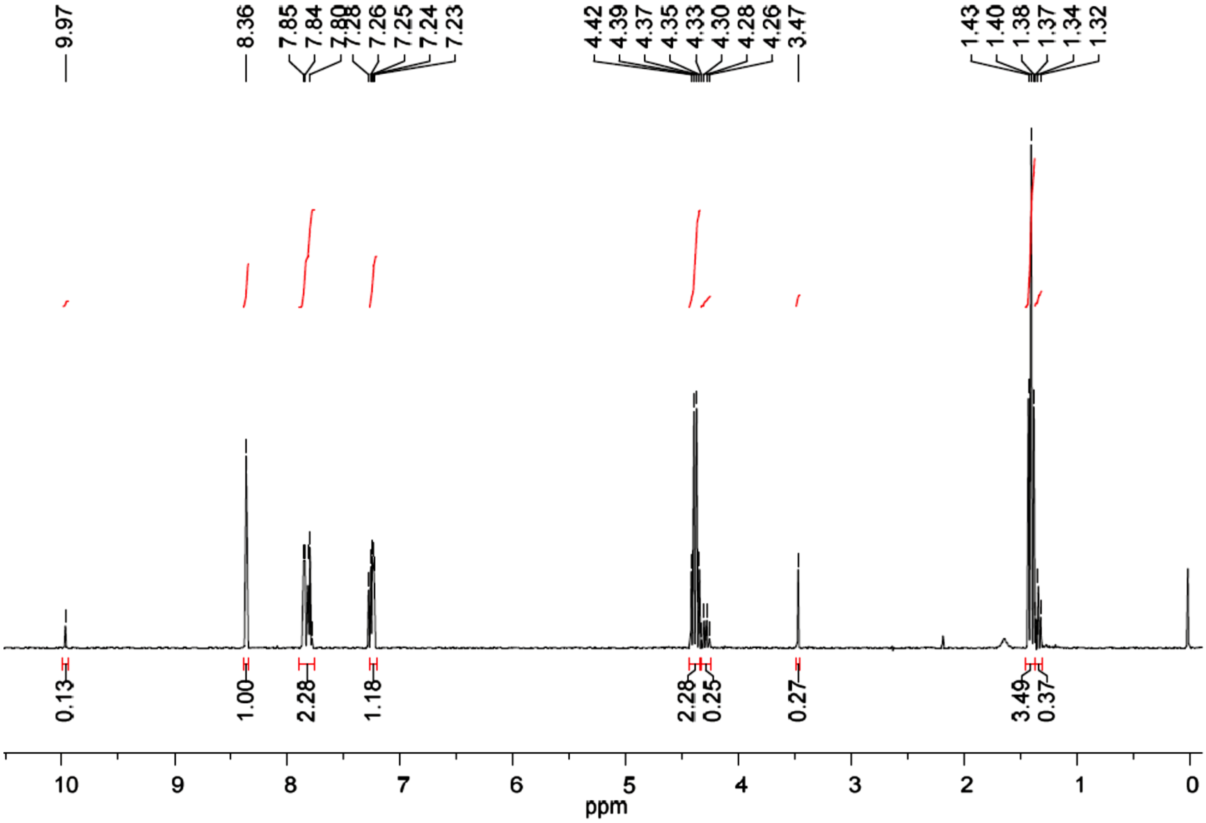

^{1}H NMR (300 MHz, $CDCl_3$) **Table 2**, **Entry 9**.

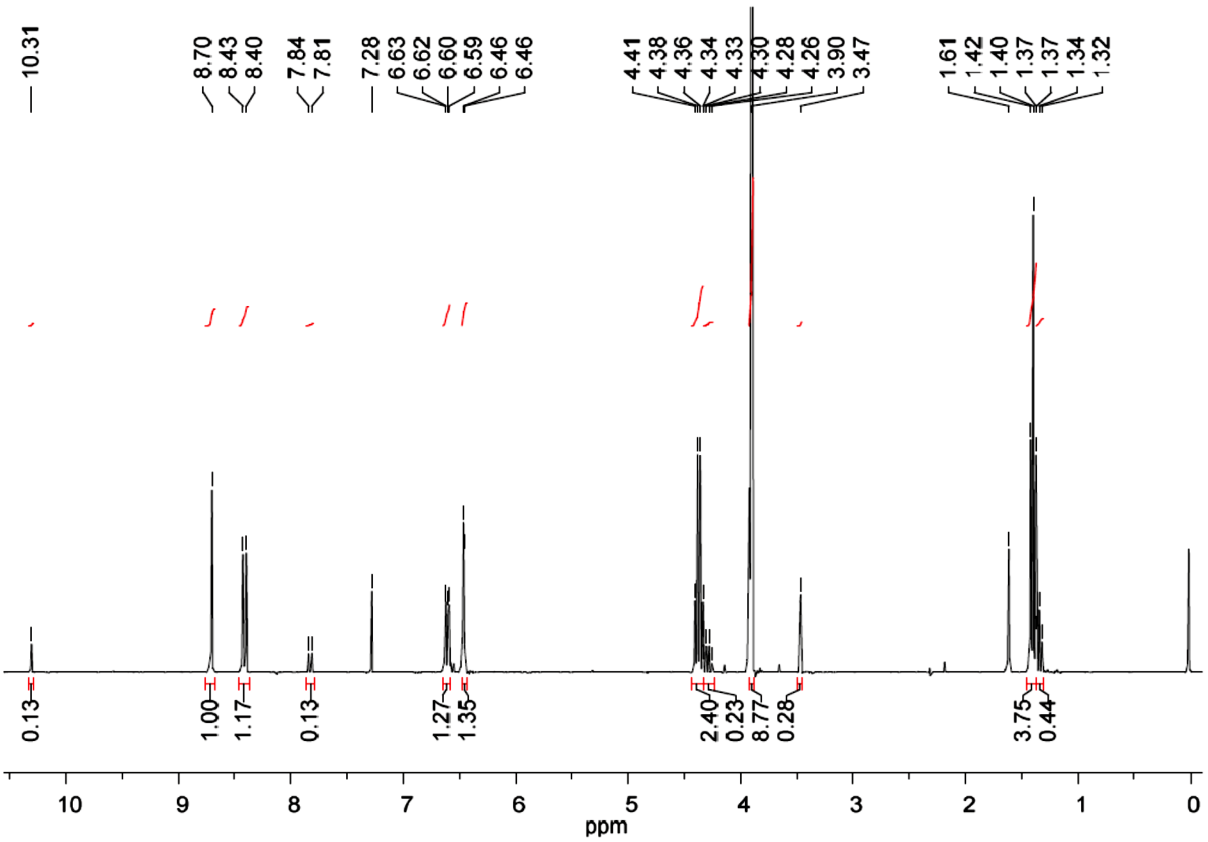

^{1}H NMR (300 MHz, $CDCl_3$) **Table 2**, **Entry 10**.

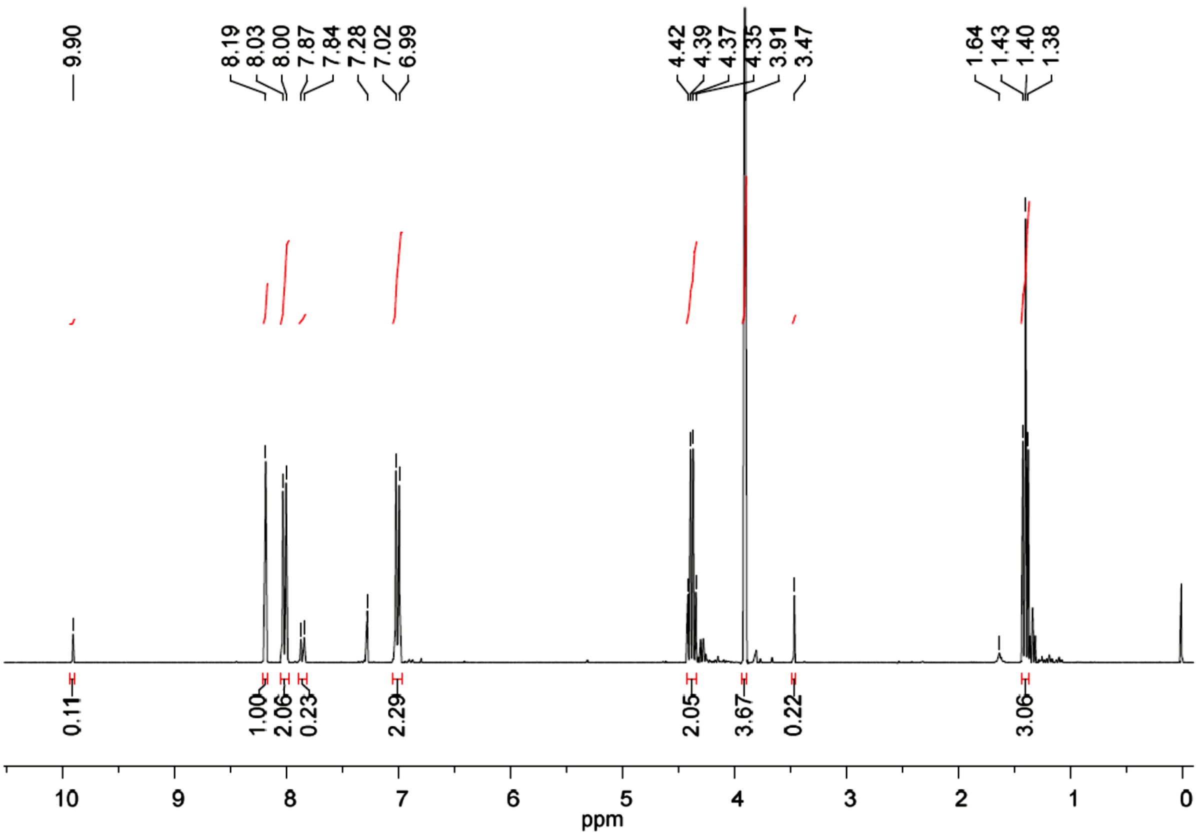

^{1}H NMR (300 MHz, $CDCl_3$) **Table 2**, **Entry 11**.

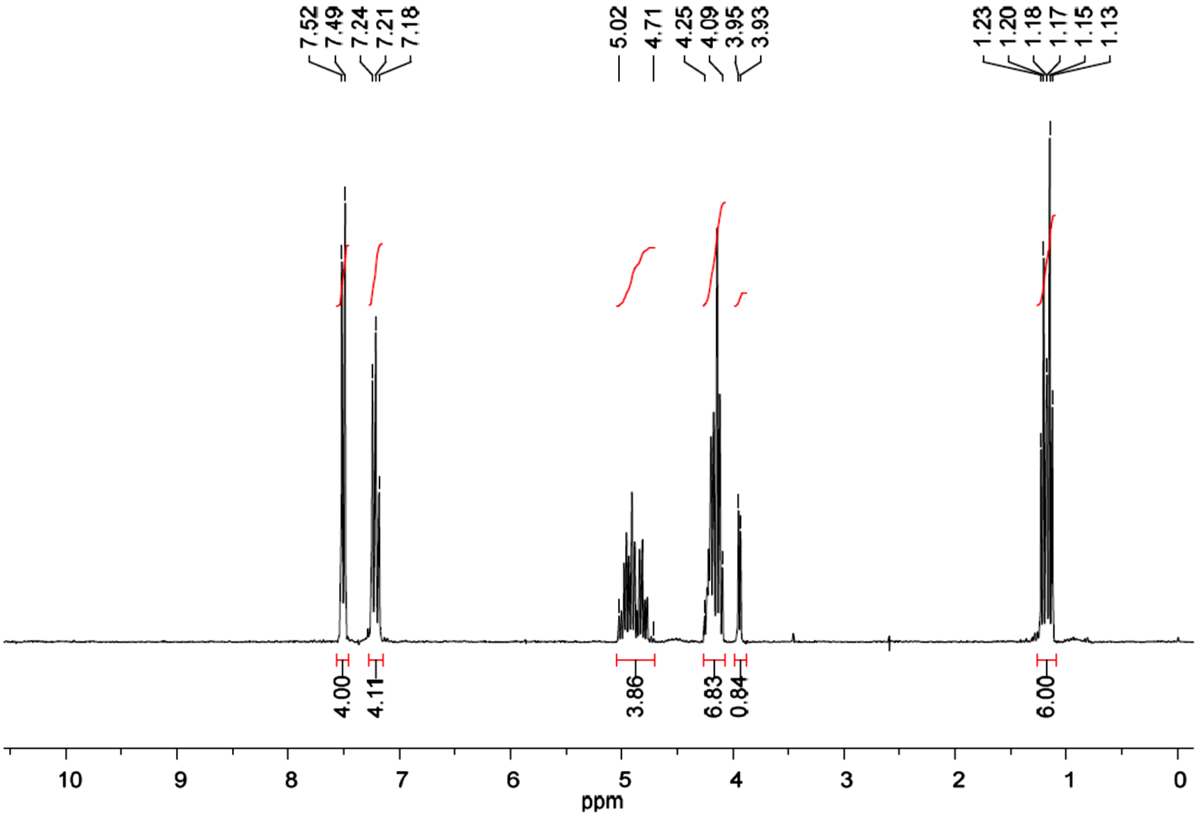

^{1}H NMR (300 MHz, $CDCl_3$) **Table 3**, **Entry 1**.

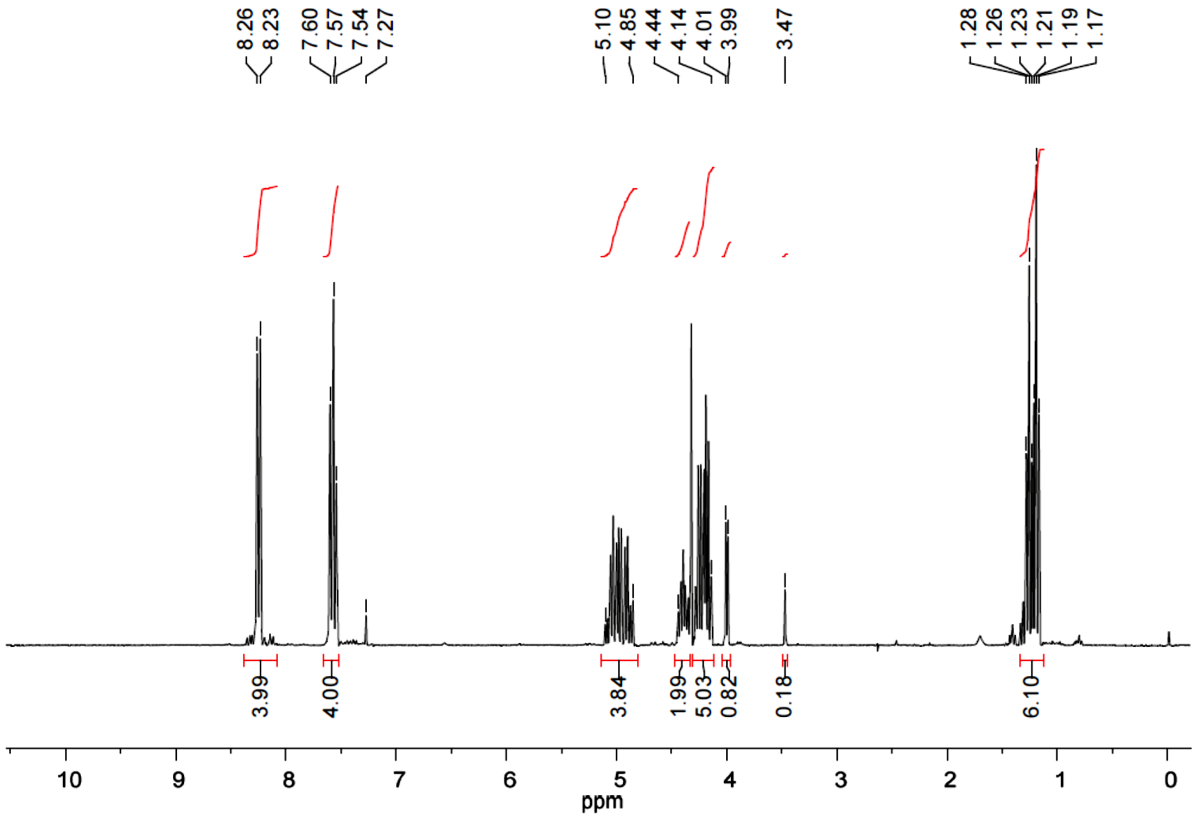

^{1}H NMR (300 MHz, $CDCl_3$) **Table 3**, **Entry 2**.

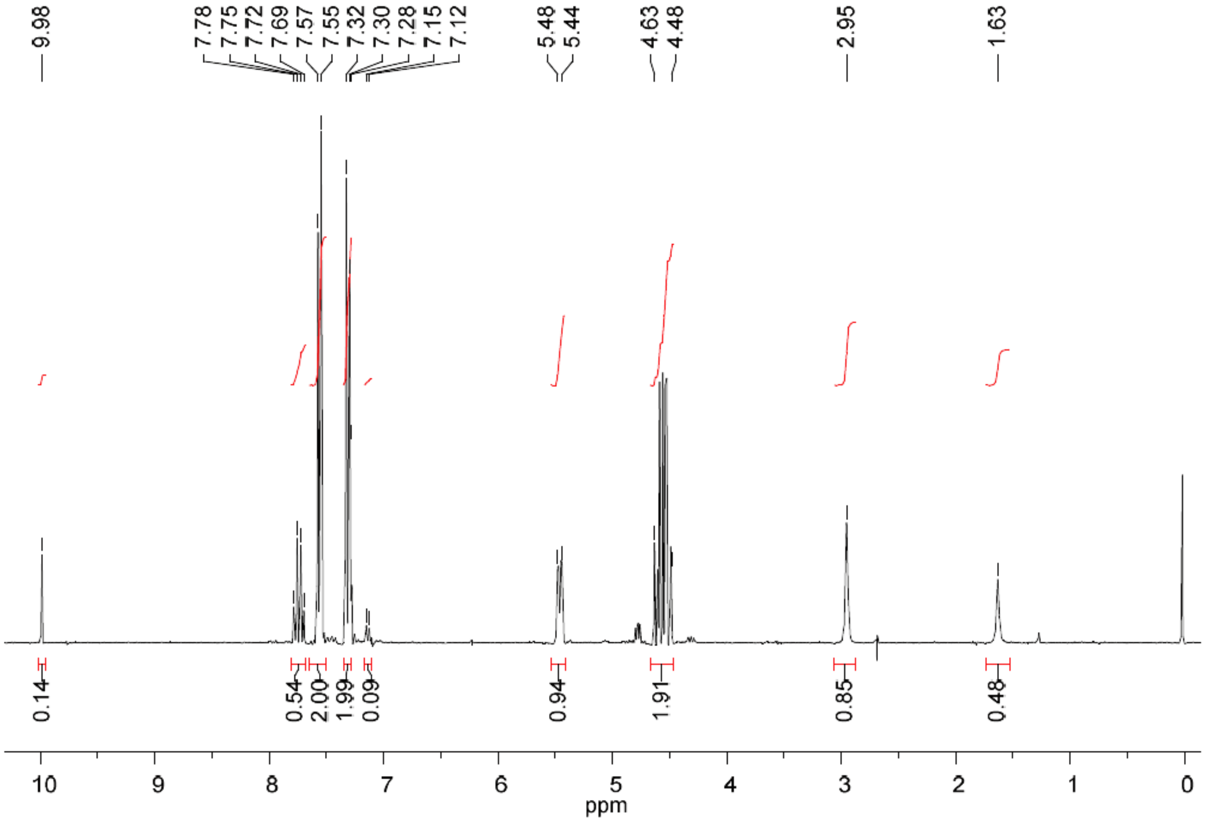

^{1}H NMR (300 MHz, $CDCl_3$) **Table 4**, **Entry 5**.

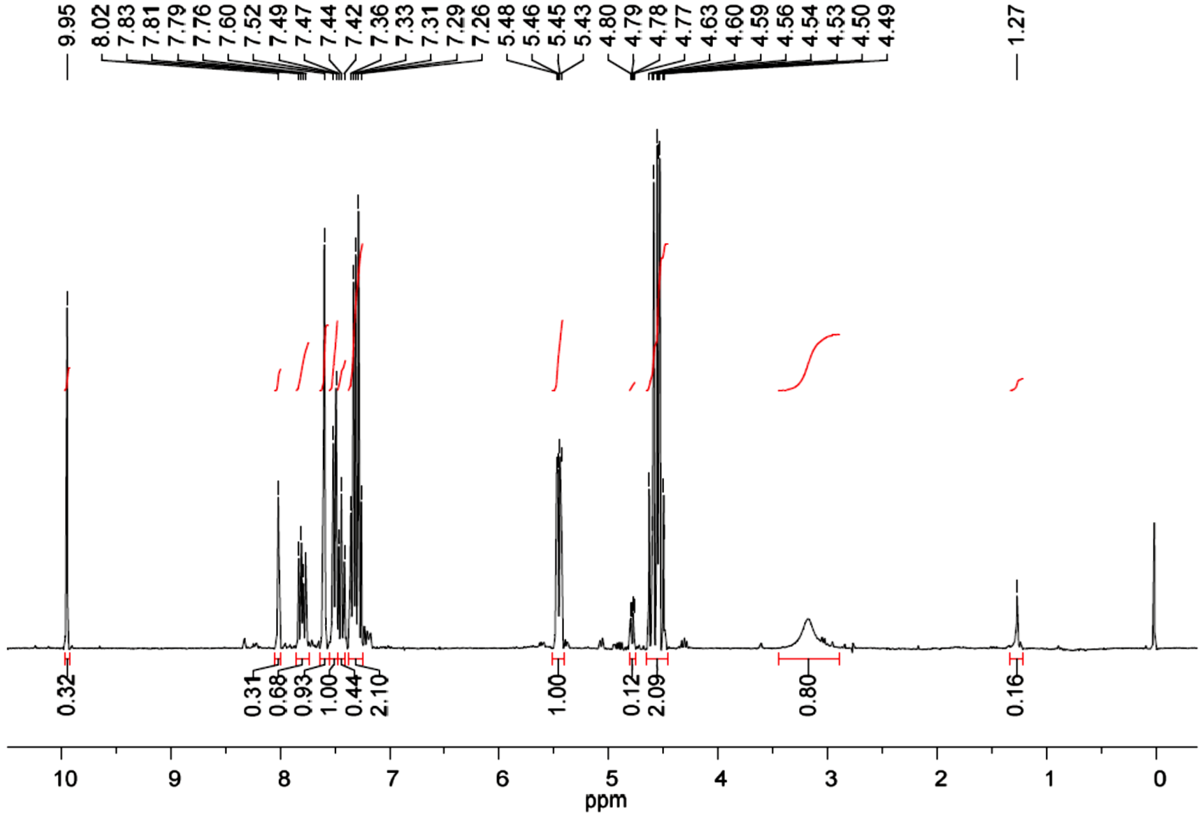

^{1}H NMR (300 MHz, $CDCl_3$) **Table 5**, **Entry 1**.

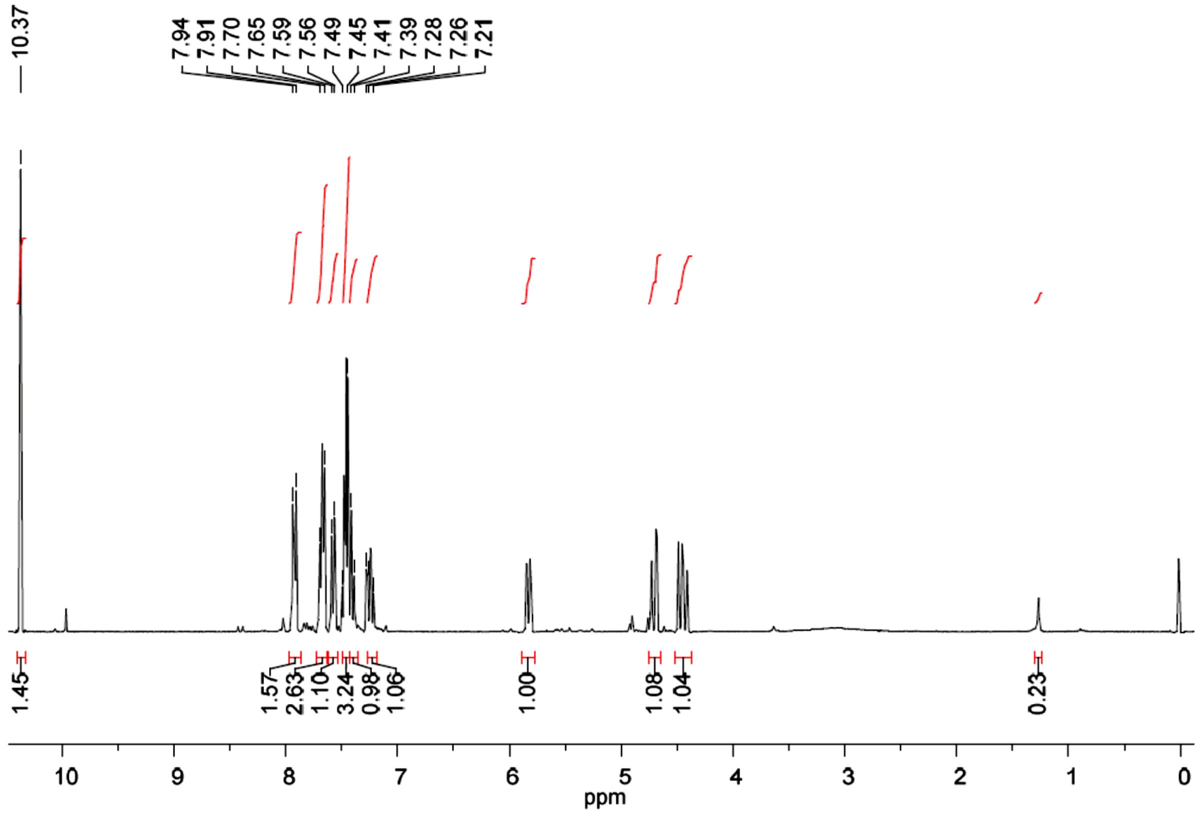

^{1}H NMR (300 MHz, $CDCl_3$) **Table 5**, **Entry 2**.

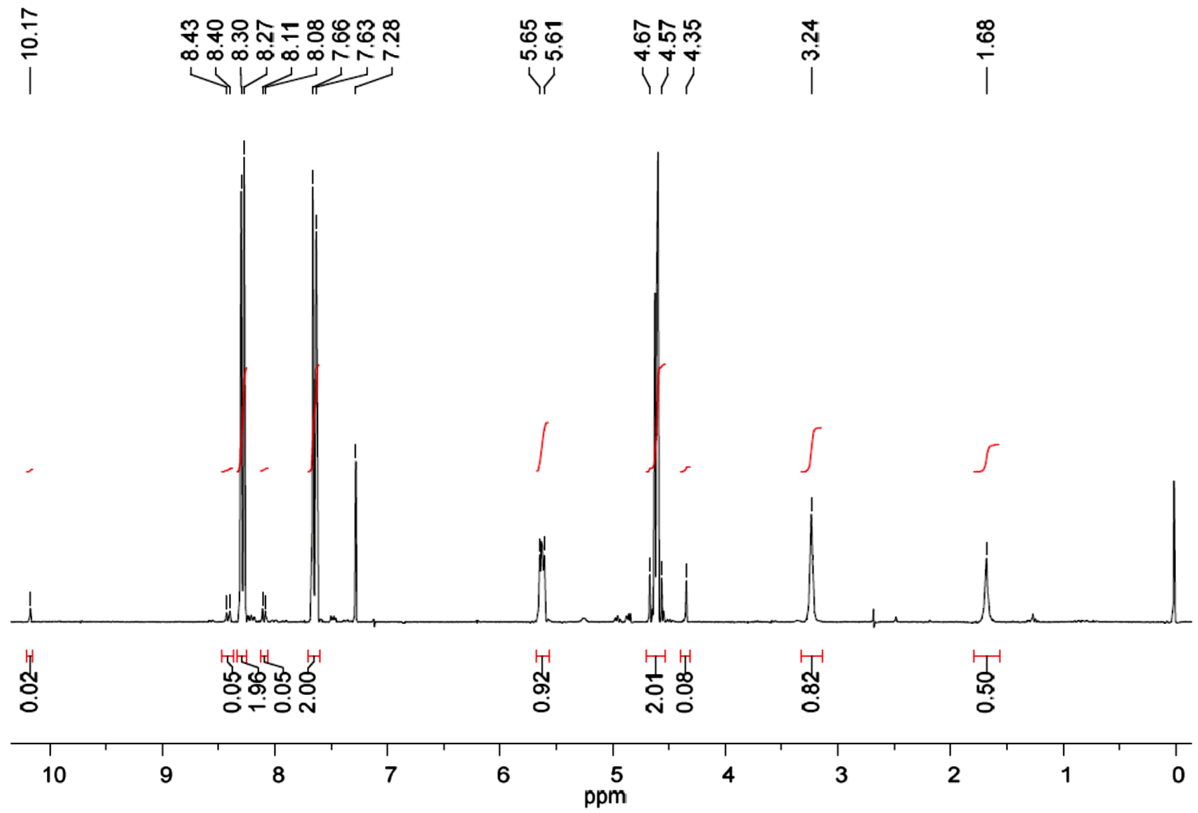

^{1}H NMR (300 MHz, $CDCl_3$) **Table 5**, **Entry 3**.

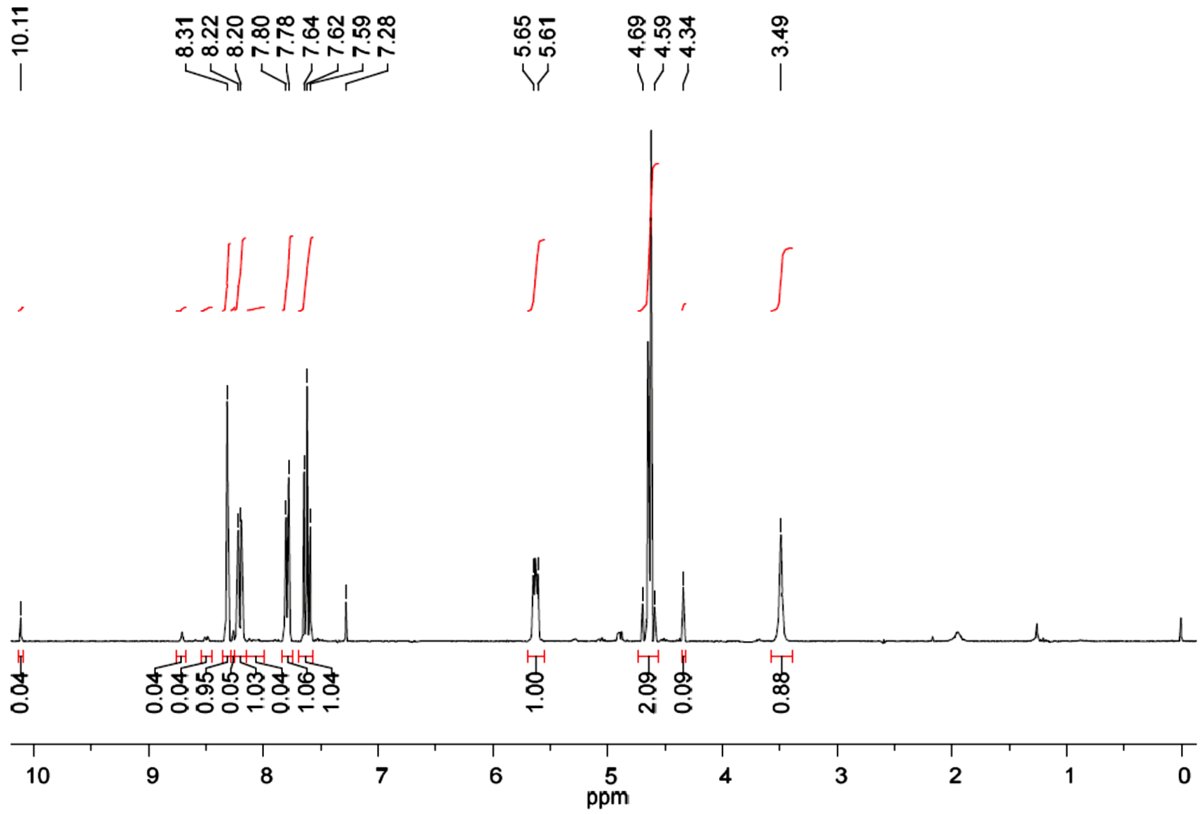

^{1}H NMR (300 MHz, $CDCl_3$) **Table 5**, **Entry 4**.

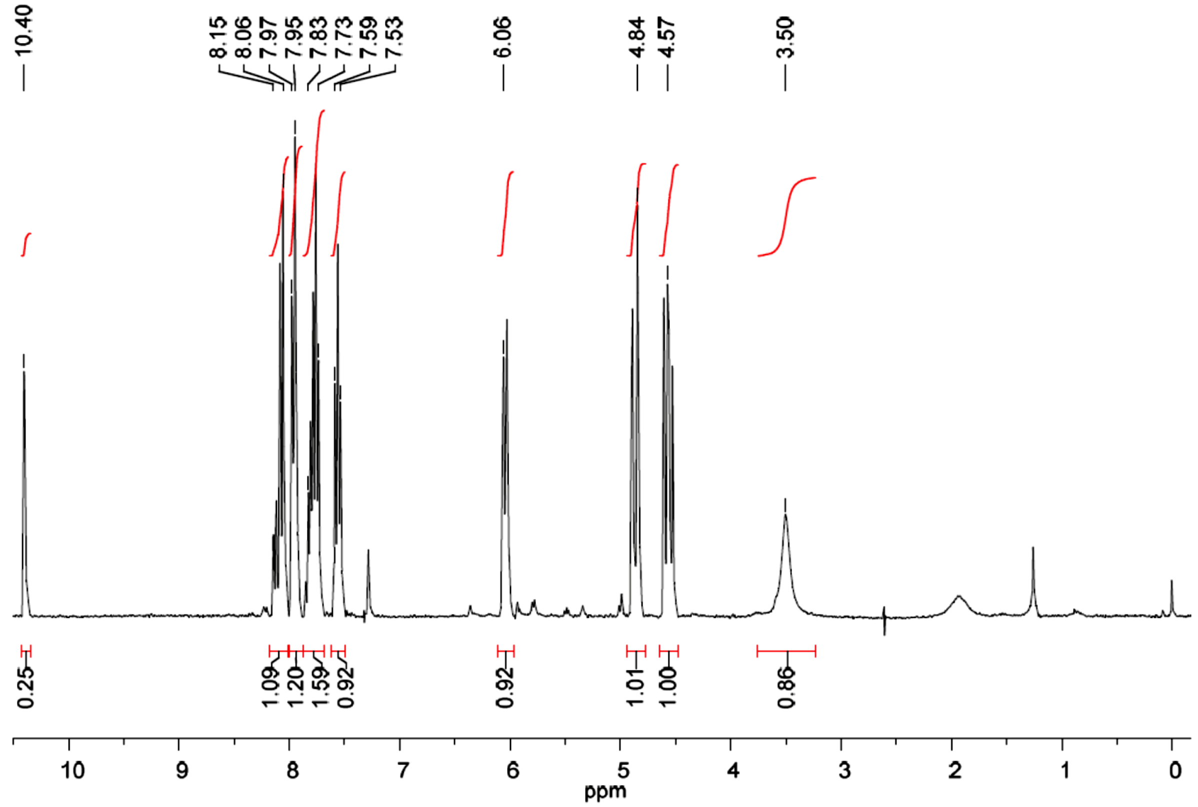

^{1}H NMR (300 MHz, $CDCl_3$) **Table 5**, **Entry 5**.

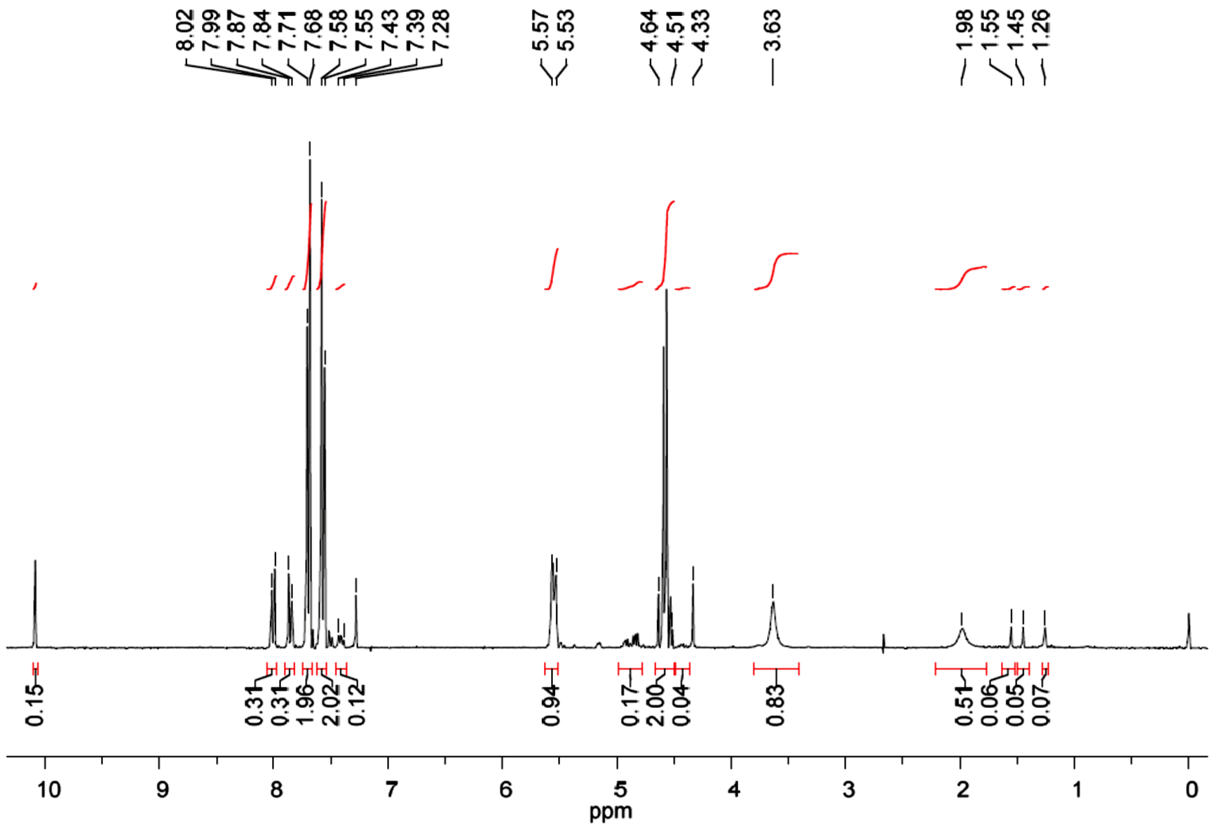

^{1}H NMR (300 MHz, $CDCl_3$) **Table 5**, **Entry 6**.

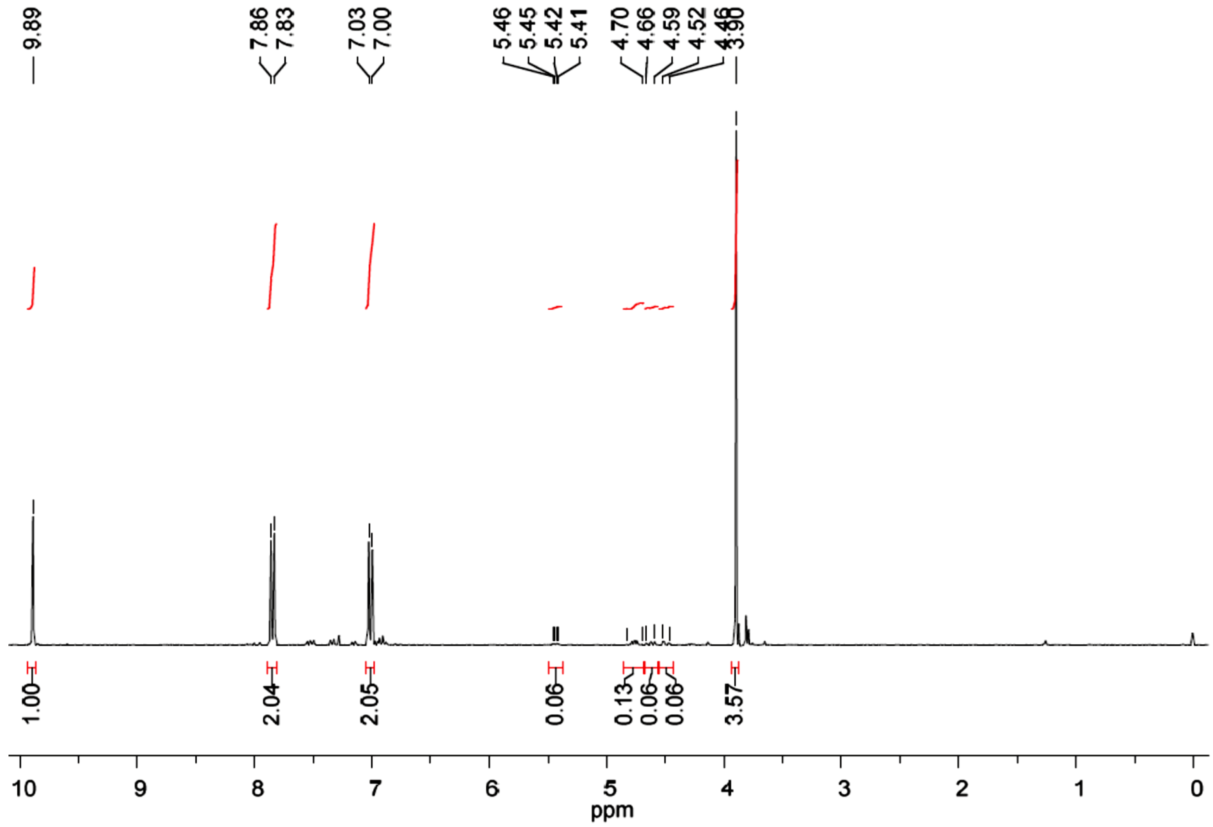

^{1}H NMR (300 MHz, $CDCl_3$) **Table 5**, **Entry 7**.

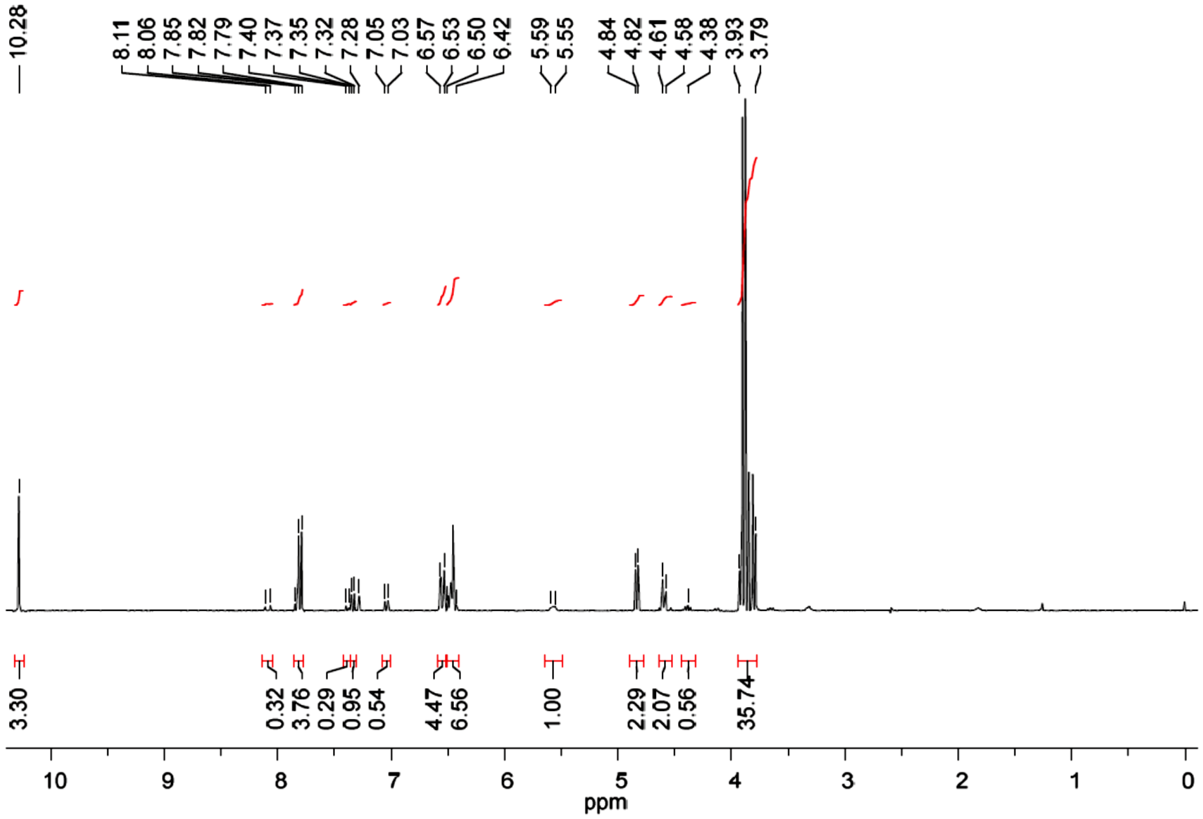

^{1}H NMR (300 MHz, $CDCl_3$) **Table 5**, **Entry 8**.
